# Distinct dynamics of neuronal activity during concurrent motor planning and execution

**DOI:** 10.1038/s41467-021-25558-8

**Published:** 2021-09-10

**Authors:** David Eriksson, Mona Heiland, Artur Schneider, Ilka Diester

**Affiliations:** 1 Optophysiology, University of Freiburg, Faculty of Biology, Freiburg, Germany; 2 BrainLinks-BrainTools, Intelligent Machine-Brain Interfacing Technology (IMBIT), University of Freiburg, Freiburg, Germany; 3 Bernstein Center Freiburg, University of Freiburg, Freiburg, Germany; 4 Present Address: Department of Physiology and Medical Physics, Royal College of Surgeons in Ireland | RCSI, Dublin 2, Ireland

**Keywords:** Motor cortex, Premotor cortex

## Abstract

The smooth conduct of movements requires simultaneous motor planning and execution according to internal goals. So far it remains unknown how such movement plans are modified without interfering with ongoing movements. Previous studies have isolated planning and execution-related neuronal activity by separating behavioral planning and movement periods in time by sensory cues. Here, we separate continuous self-paced motor planning from motor execution statistically, by experimentally minimizing the repetitiveness of the movements. This approach shows that, in the rat sensorimotor cortex, neuronal motor planning processes evolve with slower dynamics than movement-related responses. Fast-evolving neuronal activity precees skilled forelimb movements and is nested within slower dynamics. We capture this effect via high-pass filtering and confirm the results with optogenetic stimulations. The various dynamics combined with adaptation-based high-pass filtering provide a simple principle for separating concurrent motor planning and execution.

## Abstract (web summary)

Animal behaviour is governed by planning and executing movements. Here, using non-stereotypical movements to separate planning and executional processes, the authors find evidence for slow processes for planning and fast processes for movement execution.

## Introduction

Voluntary movements contain planning and execution components^1^. Execution refers to the actual movement, whereas planning is typically assumed to precede the movement. Thus, the usual way of determining preparatory neural activity relies on distinct trial phases, as in instructed movement tasks containing a delay period^2^. Using precisely timed perturbations, planning or execution roles have been assigned to specific task phases and brain areas. In particular, the delay phase between an instructive cue and an actual go-cue harbor complex processes at the border between planning and preparation of the actual motor execution. In rodents, the rostral forelimb area (RFA) and caudal forelimb area (CFA) are assigned premotor and motor roles, respectively, due to their connectivity^3^. These areas are relevant during the delay phase for subsequent movement corrections^4^. In monkeys, perturbations in the premotor cortex during this task phase delay upcoming movements^5^. These results illustrate the slightly intermingled planning and executional processes in the motor cortex. In line with this, by use of electrophysiological measurements and by contrasting the preparatory and movement activity, a controversial variety of interpretations of the role of preparatory activity has been suggested. Evidence supports the idea that, at the neuronal population level, preparatory activity represents a subthreshold activity, sharing tuning characteristics with the movement phase, but at a lower amplitude^6–12^. At the level of individual neurons, a switching of tuning has been reported between the task phases^13–16^.

To further complicate the interpretation of preparatory activity, complex individual neural responses exhibiting a variety of multiphasic patterns have been exposed during the preparatory phase^17^. A dynamic systems approach offers an option to unify these diverse findings in a common framework by describing the neuronal activity at a more abstract level^18^. To further investigate the role of preparatory activity, a differentiated approach involving various degrees of movement preparation has recently been suggested^19^. By applying three distinct task variants involving multiple degrees of movement preparation, Zimnik et al.^19^ elegantly created a gradient of decreasing motor preparation from self-initiated movements over externally cued movements to quasi-automatically generated responses. Despite this elaborate task design, a neuronal planning process (e.g., to stop an ongoing movement) can coincide and correlate with a neuronal execution process at the onset of movement, rendering a separation of both processes difficult.

This separation is particularly difficult for stereotyped movements, as there will be a correlation between the initial part of a movement and its end point. This correlation can apply to both self-initiated and instructed movements. To minimize the correlation between planning and execution, we opted for a radically different approach that does not rely on a specific task design. Instead, we allowed rats to either move freely (locomotor task) or to move a joystick in a self-paced manner (joystick task) with the sole constraint of moving as randomly as possible. With this randomized approach, the planning and executional processes at a given time will differ: The planning process will describe a future behavior that, due to the randomization, will contrast with the executed behavior. Therefore, this approach maximizes the separation between planning and executional processes using a rich repertoire of non-stereotyped movements.

To minimize the influence of a planning component on the execution activity, we separated planning from execution in terms of the lag between the neural activity and the movement^20,21^. We consider neuronal activity with a temporal behavior lag at a previously suggested range of < 100 ms^22,23^ as being related to motor execution. Moreover, we refer to neuronal activity with larger temporal behavior lags as motor planning or sensory integration, depending on whether the neuronal activity occurs before or after the movement.

**Fig. 1:**
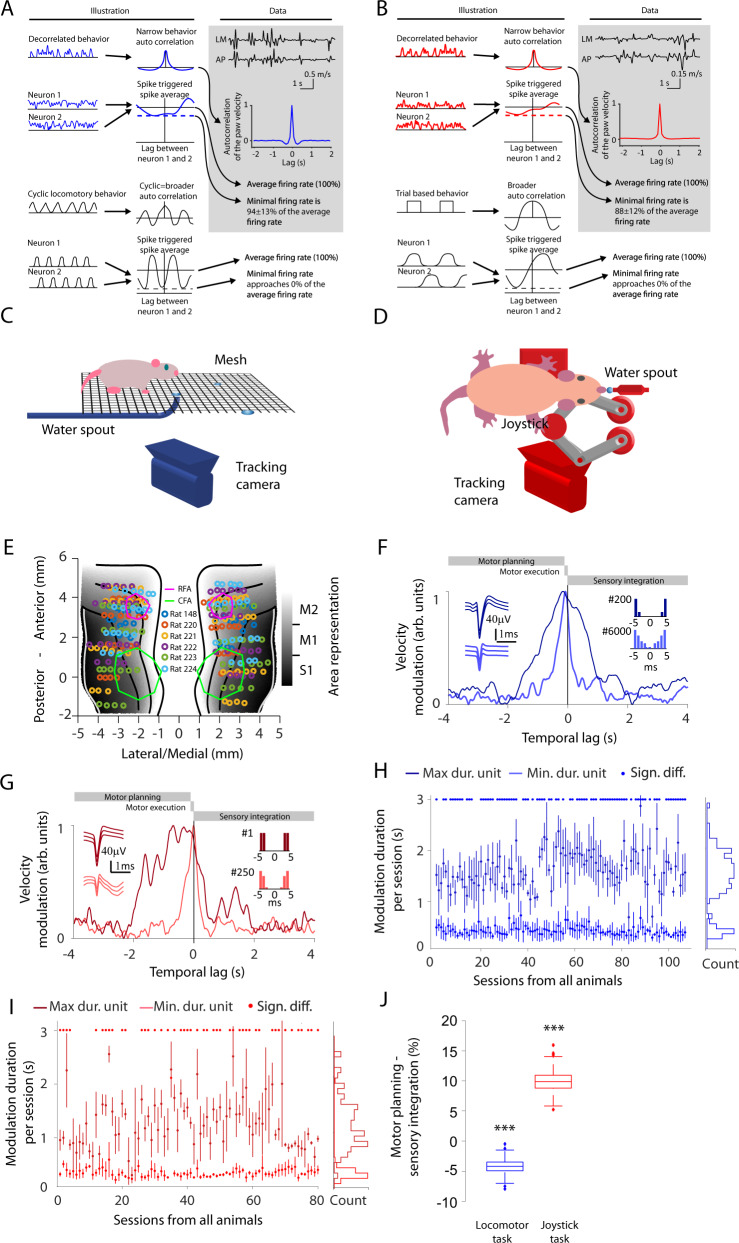
Studying neuronal dynamics with minimally repetitive behavior. **A** Illustration of the difference between decorrelated and repetitive behavior regarding behavioral autocorrelation and neuronal cross-correlation for the locomotor task. The behavioral autocorrelation is broader for repetitive locomotion (bottom panels) than for a decorrelated behavior (top panels). The minimal value (dashed line) of the neuronal cross-correlation is low if there are lags for which the two neurons do not spike (indicating correlated firing). It is high if the two neurons fire at different lags (indicating decorrelated firing) (illustration left panel). Autocorrelation for the velocity of the right front paw during the locomotor task is shown in the gray data panel. **B** Depiction of the same outline as in **A** but for the joystick task. A repeating trial structure causes correlations between different trial periods, which may, in turn, increase the width of the behavioral autocorrelation and the correlation between neurons. **C** Setup of the locomotor task. **D** Setup of the joystick task. **E** Electrode locations on the sensorimotor cortex for the respective animal. RFA and CFA are lined out by purple and green, respectively. Rostral forelimb area (RFA), caudal forelimb area (CFA), primary sensory cortex (S1), primary motor cortex (M1), and secondary motor cortex (M2). **F** Velocity modulation of the instantaneous firing rate for two example units with action potential waveforms (left inset) and interspike interval histogram (right insets) in the locomotor task. Neuronal firing rates modulated by future or past paw movement velocities are assigned to negative temporal lags (referring to planning) or to positive temporal lags (referring to sensory integration), respectively. Lags between 0 and 100 ms are considered to be related to motor execution. The dark-blue unit has a broad velocity modulation, whereas the light-blue unit is temporally precise. Both units originate from the same recording session. **G** Same outline as in **F** but for two different units in the joystick task. The dark-red unit has a broad velocity modulation, while the light-red unit is temporally precise. **H** The unit with the minimal (bright blue) and maximal (dark blue) duration of the velocity modulation for each locomotor session (*n* = 107). The error bars denote the standard deviation of bootstrapped durations. The center dot of the error bars denotes the mean of the bootstrapped durations. **I** Same outline as in **H** but for the joystick task (*n* = 80). Light red and dark red correspond to units with minimal and maximal modulation duration, respectively. **J** The summed velocity modulation for motor planning-related activity (negative lags from −1.1 to −0.1 s) minus the summed velocity modulation for sensory integration-related activity (positive lags from 0 to 1 s). Two-sided non-paired *t*-test corrected for dependence. Locomotor task: *p* = 2.2e-5, *n* = 5413, Joystick task: *p* = 2.5e-9, *n* = 2611. The data for each box-plot is bootstrapped and corrected for dependence across sessions (see Methods). On each box, the central mark indicates the median, and the bottom and top edges of the box indicate the 25th and 75th percentiles of the data, respectively. The whiskers extend to the most extreme data points, which are not considered to be outliers. Outliers are plotted individually using the “+” symbol. Significances are indicated according to ***p < 0.001. Source data are provided as a Source Data file.

This lag-based interpretation of neuronal processes is hampered by behavioral correlations. If a movement is correlated over time (e.g., because a certain pattern repeats across time), neuronal activities related to both planning and execution processes will appear to be correlated with the behavior even if a causal relationship only exists for one of the processes. A repetitive movement would cause an autocorrelation with multiple peaks (see illustration in Fig. 1A), while a prolonged behavioral state, due to reward delivery or planning, may cause one broader peak (see illustration in Fig. 1B). Broad peaks are referred to in this work as temporal bleeding. Our randomized task circumvents these problems. Based on electrophysiological recordings from rats conducting randomized movements, we characterized the relevant neuronal frequencies for planning and execution. Our data-driven approach reveals the particular relevance of a relatively low-frequency spectrum of neural changes. Note that within this low-frequency range, high frequencies are meant in a relative sense. We found a breaking point around 1.1 Hz and used this to divide the frequencies in a lower range referring to planning, and in relation to this, in a higher range contributing to the execution of movements.

Here, we show that employing minimally repetitive movements is suitable approach for separating planning, execution, and sensory processes in neuronal activity. On average, neuronal activity leads movements in a trained joystick task whereas neuronal activity succeeds movements in a non-trained locomotor task. Using this approach of minimally repetitive movements we demonstrate that processes are slower towards secondary motor areas and that those slower processes also precede movements by longer latencies. We describe how the movement onset is preceded by an increase in the frequency of neuronal activity dynamics and how these preceding dynamics become temporally coordinated across neurons shortly before movement onset. These findings propose coordinated high-frequency changes in neuronal activity as one aspect of executing movements and slow frequency changes as a way to integrate information and plan upcoming movements.

## Results

### Minimally repetitive movement tasks

To reduce the temporal bleeding between planning and execution, we aimed to minimize correlations by encouraging animals to conduct movements with a minimal recurrence of individual movement sequences in two different settings. One task was based on walking (locomotor task) and the other was based on joystick movements (joystick task). In the locomotor task, rats moved unconstrained in a box while searching for pseudo-randomly placed water drops on a floor mesh (Fig. 1C). In this task, neither the overall movement (Supplementary Fig. 1A) nor the directedness of the movement was changed across sessions (Supplementary Fig. 1B). In the joystick task, we trained rats to move a joystick with their right front paw while minimizing the revisiting of previously occupied positions (Fig. 1D). For the joystick task, the overall movement range increased across sessions (Supplementary Fig. 1A), and rats learned to explore the anterior-posterior movement direction in later sessions (Supplementary Fig. 1B). Therefore, only recordings after 70 h of training in the joystick task were used for data analysis. In both tasks, movements were not repetitive, as is indicated by narrow temporal behavioral autocorrelations of the movement velocities (see data boxes in Fig. 1A, B). To evaluate the typical movement duration, we calculated the mean and SEM of the frequency velocity spectrum (locomotor task: 4.7 ± 1.05 Hz, joystick task: 4.3 ± 0.12 Hz). These frequencies refer to the time between two peak velocities (with a trough velocity in between), which equals 1/frequency and represents the movement duration (210 ms in the locomotor task, 230 ms peak in the joystick task).

To study the neuronal underpinnings of decorrelated movements, we trained six Long–Evans rats in the locomotor task. Five of these six animals were also trained in the joystick task. To record neuronal activity, electrodes were placed bilaterally in the sensorimotor cortex (42 electrodes per animal, Fig. 1E). We targeted the output layer V by implanting the electrodes at a depth of 1.2 mm^24,25^. In total, we recorded 5691 single units (SU) and 7325 multi-units (MU) over 107 sessions for the locomotor task (Supplementary Table 1). For the joystick task, we recorded 4041 SU and 5268 MU over 80 sessions (Supplementary Table 2). We refer to SU and MU collectively as sorted units.

We first examined whether the movement decorrelation showed up in the statistics of the neuronal activity. For repetitive behavior, neurons may fire at a specific lag relative to each other, causing some lags to be less represented than others. This imbalance causes the firing rate for some lags to be fundamentally lower than the average firing rate (see dashed lines in Fig. 1A, B). Neuronal activity was characterized by a decorrelated pair-wise spiking; that is, pairs of neurons fired independently such that all lags were equally represented and the firing rate at a certain lag was close to the average. The firing rates of one neuron relative to another at the least-represented lag were 94 ± 13% and 88 ± 12% of the average firing rate for the locomotor and joystick task, respectively (Fig. 1A, B, see Methods). These results are indicative of decorrelated neuronal activity.

### Temporal precision

The temporal decorrelation of both the behavioral and the neuronal activity maximizes the temporal precision of the estimated functional relationship between movement and neuronal activity. To quantify the temporal precision, we calculated the range of temporal lags for which a given sorted unit was modulated by the paw velocity via a lookup table (Fig. 1F, G and Supplementary Fig. 2). We refer to this modulation across lags as velocity modulation. Furthermore, we use the term modulation duration to refer to the duration at which the velocity modulation exceeded 80% of the peak modulation. We observed units with both long modulation durations (locomotor task: 1.6 ± 0.34 s, joystick task: 1.2 ± 0.32 s) and short modulation durations (locomotor task: 0.36 ± 0.15 s, joystick task: 0.31 ± 0.09 s) within the same session (Fig. 1H, I). This observation demonstrates that our approach minimized behavioral bleeding to an extent permitting the separation of long processes, such as motor planning and sensory integration, from shorter processes such as motor execution.

Finally, this behavioral approach enabled us to quantify the relative strength of motor planning and sensory integration, by taking the normalized difference of the velocity modulation for negative and positive temporal lags. In line with previous lesion and inactivation approaches^26–29^, the relative contribution of the motor planning-related activity was larger for the joystick task (9.9 ± 1.7%, mean ± SEM, *p* < 0.0001, two-tailed *t*-tests), while the sensory integration-related activity was larger in the locomotor task (−4.2 ± 1%, mean ± SEM, *p* < 0.0001, two-tailed *t*-tests, Fig. 1J and Supplementary Fig. 1C). For the joystick task, the motor planning-related activity increased relative to the sensory integration-related activity for later training sessions (Supplementary Fig. 1C). Thus, our approach based on minimal repetitive movements complements previous studies with a temporally refined neuronal activity-based assay of the gradient from motor planning and execution to sensory integration for skilled and locomotor behavior.

### Varying neuronal modulation durations

**Fig. 2:**
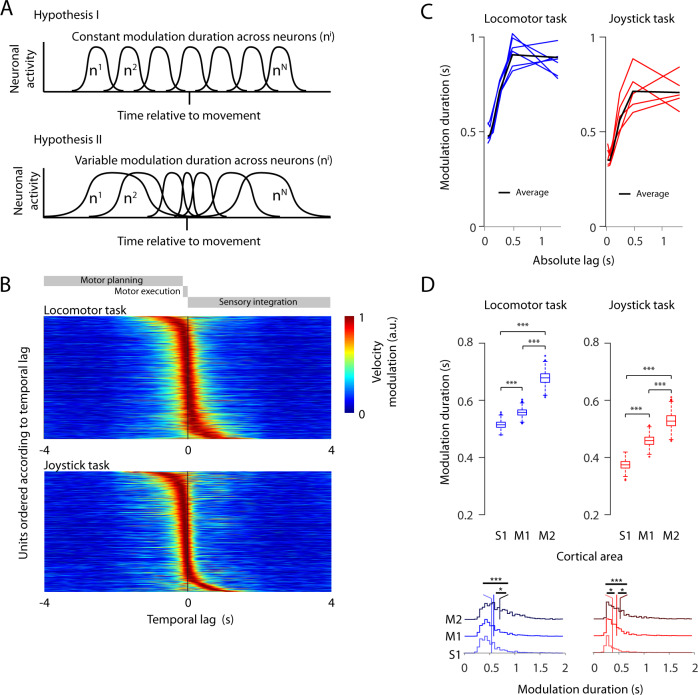
The duration of velocity modulation of individual units depends on the temporal lag to behavior and on the cortical area. **A** Two hypotheses of sequential neuronal activity relative to movement. The duration of the neuronal activity can be constant across lags (upper panel) or different across lags (lower panel). **B** Units sorted according to the lag of their maximum velocity modulation in the locomotor task (top), and for the joystick task (bottom). **C** Relationship between average modulation duration and temporal lag (black line), across the different animals (colored lines) for the locomotor (left) and joystick task (right). **D** Top panel: Duration of the velocity modulation for each cortical area for the locomotor task (left) and the joystick task (right). Two-sided non-paired *t*-test corrected for dependence (see methods). Locomotor task: S1 (*n* = 1766), M1 (*n* = 2450), M2 (*n* = 1197), M1-M2: *p* = 1.7e-35, S1-M2: *p* = 3.7e-53, S1-M1: *p* = 1.3e-9. Joystick task: S1 (*n* = 837), M1 (*n* = 1015), M2 (*n* = 759), M1-M2: *p* = 3.9e-9, S1-M2: *p* = 2.1e-31, S1-M1: *p* = 2.8e-17. The data for each box-plot is bootstrapped and corrected for dependence across sessions (see methods). Box-plots: central mark indicates the median, bottom and top edges refer to the 25th and 75th percentiles. Primary sensory cortex (S1), primary motor cortex (M1), and secondary motor cortex (M2). Bottom panel: Histograms of the modulation duration for each cortical area for the locomotor task (left) and the joystick task (right). A mixed-effect model was used to calculate significances between the cortical areas. Mixed-effect model (see methods): Locomotor task: M1-M2: *p* = 2.2e-6, S1-M2: *p* = 4.0e-5, S1-M1: *p* = 0.23. Joystick task: M1-M2: *p* = 0.0142, S1-M2: *p* = 6.2e-5, S1-M1: *p* = 0.016. Significances are indicated according to *p < 0.05, **p < 0.01, and ***p < 0.001. Source data are provided as a Source Data file.

For the example units in the joystick task, we noted that the velocity modulation increased earlier for units with longer velocity modulations compared to units with a short velocity modulation (Fig. 1G). Motivated by this difference, we examined whether the modulation duration across units was independent of the temporal lag (Fig. 2A, upper panel) or, alternatively, increased with larger temporal lags relative to the movement (Fig. 2A, lower panel). We defined a temporal lag based on the peak of the velocity modulation (see Methods). In accordance with the second hypothesis, the modulation duration increased significantly with increasing temporal lags for both locomotor and joystick tasks (analysis of variance (ANOVA), locomotor task, *p* < 0.0001, ANOVA joystick task, *p* < 0.0001, Fig. 2B, C). The longer velocity modulation for larger lags is not due to a larger temporal scatter, as the variability of the velocity modulation was not increasing with increasing temporal lag (Pearson correlation, locomotor task, *p* > 0.05, joystick task: *p* > 0.05, Supplementary Fig. 3A). To test whether the slower dynamics (indicated by longer modulation durations) were the result of slow balancing head and shoulder movements, we calculated the neck velocity modulation duration in the locomotor task. The average neck velocity modulation duration was not larger than that of the paw movements (Supplementary Fig. 1B), and the peak of the neck velocity modulation did not appear earlier than that of the paw movements (Supplementary Fig. 1C). These facts rule out neck movements as the main instigators of slower neuronal dynamics. Instead, the results suggest that a putative motor execution represented by units with shorter temporal lags occurred with faster neural dynamics than motor planning and sensory integration.

### Integration timing of cortical areas

In rodents, the premotor and motor cortex have been defined; that is, the secondary motor cortex (M2) is RFA, and the primary motor cortex (M1) is CFA^3,30,31^. In contrast to M1, M2 has been assigned both motor and higher-order functions^32–36^ and might be related to the premotor cortex of primates^3,35,37–39^. Similar to studies in non-human primates, a hierarchical relationship has been observed during movement control in rats, with M2 leading M1^40,41^. This hierarchy influences the accuracy of a movement^42^ and is involved in the integration of motor information with internal state information for an adaptation to goal-directed behaviors^43^. If motor planning and sensory integration are associated with longer modulation durations, it is conceivable that a higher brain area, such as M2 (which presumably is functionally similar to the premotor cortex in primates) contains neurons with longer modulation durations than M1. To test this, we mapped the electrode locations onto the nonlinear gradient spanning M2, M1, and the primary somatosensory cortex (S1) (Fig. 1E). Indeed, neurons in higher areas (i.e., M2) had a significantly longer modulation durations than neurons in lower areas (i.e., M1 and S1, Fig. 2D). This observation was true for both the locomotor and the joystick task (ANOVA, locomotor task: *p* < 0.0001; linear mixed-effect model: S1 vs. M1, *p* = 0.23, M1 vs. M2, *p* < 0.0001, S1 vs. M2, *p* < 0.0001; joystick task: *p* < 0.0001; linear mixed-effect model: S1 vs. M1, *p* = 0.015, M1 vs. M2, *p* = 0.014, S1 vs. M2, *p* < 0.0001). On average, neurons in S1, M1, and M2 had modulation durations of 512 ± 14, 557 ± 13, and 677 ± 22 ms, respectively, during the locomotor task, and 371 ± 16, 456 ± 18, and 526 ± 26 ms (mean ± SEM), respectively, during the joystick task. The results in this and the previous section indicate that slower changes in neuronal activities govern non-movement-related processes such as planning. Faster changes in neuronal activities govern primary processes such as motor execution.

### Population activity changes more quickly during movement

**Fig. 3:**
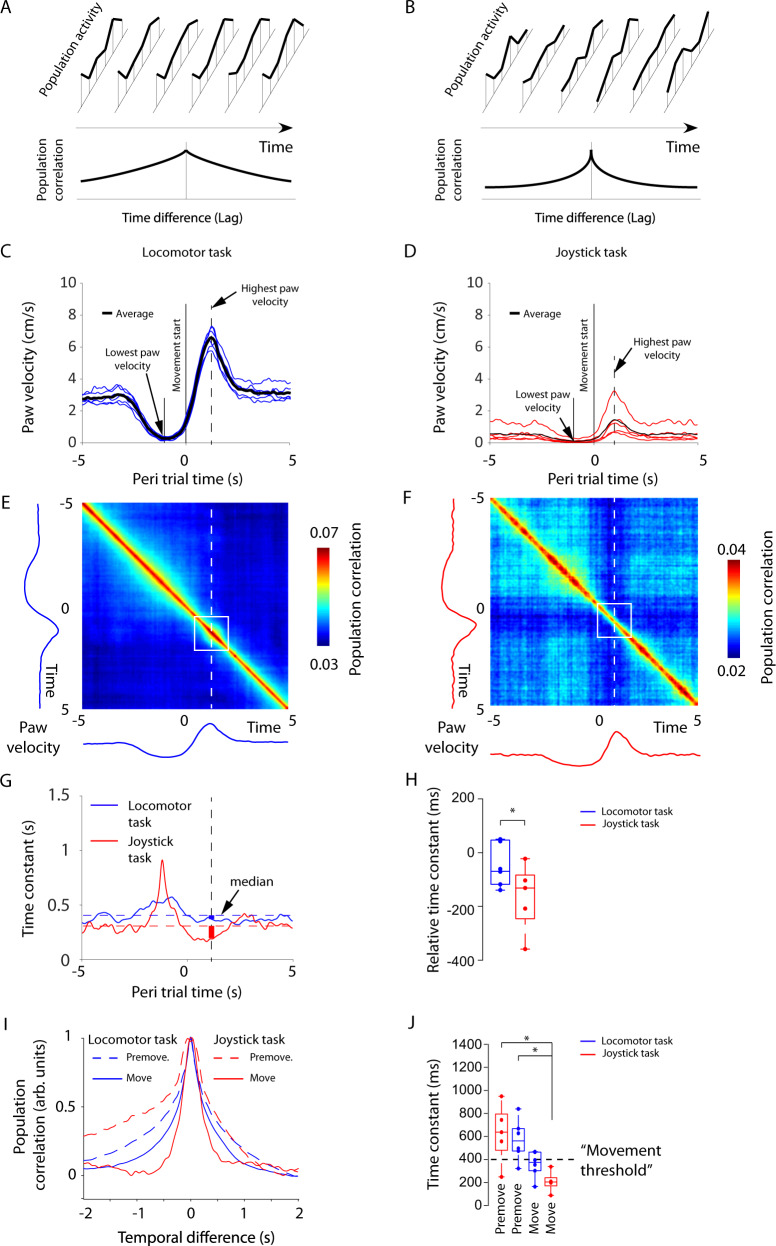
The population activity changes faster during motor execution in the joystick task than in the locomotor task. **A** Illustration of how slowly changing population activity leads to temporally broader population correlations. **B** Same as in **A**, but for faster-changing population activity. **C** Average paw velocity across behavioral trials for the locomotor task (see Methods for the trial definition). Blue lines denote data from individual animals and the black line denotes the average across all animals. **D** Depiction of the same outline as in **A** but for the joystick task. **E** Average pair-wise correlations of population vectors across all animals for the locomotor task. **F** Outline as in **C** but for the joystick task. **G** The time constant of the decay in the population correlation across the trial in the locomotor task (blue) and the joystick task (red). The median relative time constants are included as dotted lines. **H** The decrease in time constant during movement in relation to the median time constant across the trial for locomotor and joystick task. Two-sided non-paired *t*-test *p* = 0.04 (Joystick task: *n* = 5 and Locomotor task: *n* = 6). Box-plots: central mark indicates the median, bottom and top edges refer to the 25th and 75th percentiles. Points denote individual animals. **I** Decay in population correlation at the premovement time point (at −1 s during lowest paw velocities) and at the movement time point (at 1 s during highest paw velocities). **J** Time constants for the locomotor and joystick task for the premovement and movement time points based on the curves in **G**. Joystick task premove – Joystick task move two-sided non-paired *t*-test *p* = 0.0348 (*n* = 5). Locomotor task premove – Joystick task move two-sided non-paired *t*-test *p* = 0.0108 (*n* = 6, and 5). Bonferroni corrected for multiple comparisons: premove versus move (4 different possibilities). Box-plots: central mark indicates the median, bottom, and top edges refer to the 25th and 75th percentiles. Points denote individual animals. Significances are indicated according to *p < 0.05. Source data are provided as a Source Data file.

Next, we examined whether the neuronal activity changed at a higher rate during movement execution than during putative motor planning and whether this change was particularly fast during trained behavior, such as the joystick task. As the spiking activity of individual units contained a large variability, we tested the rate of change in terms of the population activity (Fig. 3A, B). To this end, we correlated the population activity (across all sorted units in one session) between two time points of various temporal distances ranging between 0 and 10 s with a resolution of 10 ms. This population correlation will typically decrease with the increasing temporal distance between the two time points. The rate of decay is quantified by the time constant of an exponential fit. This population correlation decay is a measure of the frequency characteristics of the population dynamics: One over the time constant defines the frequency at which a low-pass filter with that time constant attenuates the amplitude to 16% of the original amplitude.

To compare the time constant during movement and behavioral quiescence, we defined trials between the time point of lowest paw velocity, which we refer to as premovement, and the time point of highest paw velocity, which we refer to as movement (see Methods, Fig. 3C, D). Although the population correlation followed a similar motive, with a less-confined diagonal during premovement and a more-confined diagonal during movement, robust bands of low correlation during movement execution occurred in the joystick task but not in the locomotor task, thus revealing a qualitatively different correlation structure (Fig. 3E, F). These bands of low correlation are a sign of a brief time constant, indicating that the population activity changed rapidly during motor execution.

During periods of movement, population correlations decayed significantly faster than the median time constant in the joystick task (−176 ± 59 ms, mean ± SEM, *n* = 5, *p* = 0.043, two-tailed *t*-tests) but not in the locomotor task (−18 ± 27 ms, mean ± SEM, *n* = 6, *p* = 0.54, two-tailed *t*-tests, Fig. 3G, H). In line with the strong decrease in time constant in the joystick task during movements (Fig. 3I), the time constant during joystick movements was lowest (203 ± 88 ms, mean ± SEM, *n* = 5, Fig. 3J) indicating a faster-changing population activity. In contrast, the time constant was largest (i.e., the population activity was stable) during joystick premovement periods, which presumably involves motor planning (761 ± 375 ms, mean ± SEM, *n* = 5, Fig. 3J). The difference in the time constants cannot be explained by behavioral differences across the two tasks (summarized in Supplementary Note 1 and Supplementary Fig. 4). Across areas, the time constant only fell below the baseline level for S1 for the locomotor task (although this drop was not significant), and across all areas, S1, M1, and M2 for the joystick task (Supplementary Fig. 5).

Finally, to rule out the possibility that the observed frequency gating was only valid for fast-movement changes, we tested how the time constant depended on the instantaneous frequency of the movements (see Methods, Supplementary Fig. 6). The decrease in correlation followed an exponential decay for both the locomotor task and the joystick task, indicating that the time constant is a good proxy for the frequency content of the neural dynamics. For the joystick task, apart from a slight increase in the lowest movement frequency, the neural time constant was short and independent of the movement frequency (*τ* = 0.32 to 0.4 s). In contrast, the neural time constant for the locomotor task was longer (*τ* = 0.66 to 0.69 s) and only decreased for the highest movement frequency (*τ* = 0.36 s).

The faster decay in the joystick task compared to the locomotor task corroborates the idea that fast processes are mainly involved in movement execution. Referring to the finding that the joystick task involves a trained component (see Supplementary Fig. 1), behaviors relying on training typically require the participation of the motor cortex^26^. This point is in concert with the notion that short velocity modulations are associated with faster changes for processes that start immediately before the movement and are therefore related to the movement’s execution. In contrast, the slowly changing activity in the locomotor task, which relies less on the participation of the motor cortex^44^, may be related to a mix of planning, execution, and sensory signals. This suggestion aligns with the observation that longer velocity modulations are associated with slower changes for processes that start well before movement, such as planning.

### Fast changes in neuronal activity precede movement

**Fig. 4:**
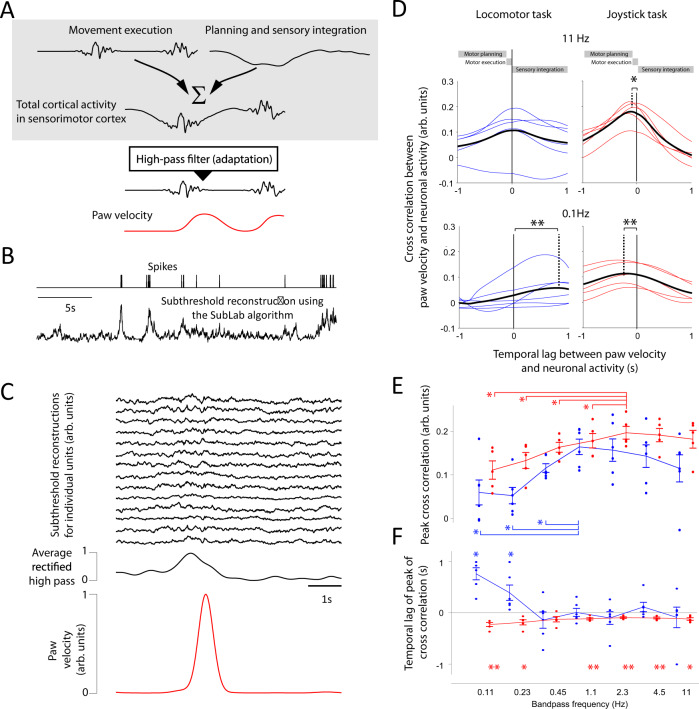
High-pass-filtered neural activity is correlated to paw velocities. **A** Schematic illustration of how a high-frequency neuronal activity can be superimposed on a low-frequency neuronal activity and yet be separable. **B** Reconstruction of the subthreshold activity of the sorted units to enable the study of how fast neuronal activities change. **C** Reconstruction of neuronal activities from 14 randomly selected units during the joystick task (top). An increase in the average rectified high-pass-filtered neuronal activity (black trace, middle row) typically precedes higher paw velocity (red trace). **D** The Pearson correlation coefficient for different lags between the rectified band-pass (11 Hz)-filtered neuronal activity and the paw velocity during the locomotor task (upper-left, two-sided *t*-test, *p* = 0.7, *n* = 6), and the joystick task (upper right, two-sided *t*-test, *p* = 0.0115, *n* = 5), and for recitified band-pass (0.11 Hz)-filtered neuronal activity and the behavior during the locomotor task (lower left, two-sided *t*-test, *p* = 0.0013, *n* = 6), and the joystick task (lower right, two-sided *t*-test, *p* = 0.0051, *n* = 5). The comparison between cross-correlation values at time point zero and the time point of maximal cross-correlation reveals significant changes with different temporal lags. **E** The peak correlation between the paw velocity and the rectified band-pass-filtered neuronal activity for seven frequencies for the joystick task (red), and the locomotor task (blue). Two-sided paired *t*-test against the peak at 1.1 Hz for the locomotor task: *n* = 6. *p* = 0.016 (0.11 Hz), 0.0029 (0.23 Hz), and 0.019 (0.45 Hz), and against the peak at 2.3 Hz for the joystick task: *n* = 5. *p* = 0.036 (0.11 Hz), 0.012 (0.23 Hz), 0.0085 (0.45 Hz), and 0.021 (1.1 Hz). No correction for multiple comparisons. Lines connecting the data points (denoting individual animals) across the frequencies denote the mean for the locomotor task (blue), and for the joystick task (red). Error bars denote the standard deviation of the mean. **F** Temporal lags of the peak Pearson correlation coefficient (across temporal lags) for rectified band-pass-filtered neuronal activity. Two-sided paired *t*-test against the 0 s lag for the locomotor task: *n* = 6. *p* = 0.0013 (0.11 Hz), and 0.045 (0.23 Hz), and for the joystick task: *n* = 5. *p* = 0.0051 (0.11 Hz), 0.025 (0.23 Hz), 0.0056 (1.1 Hz), 0.005 (2.3 Hz), 0.0072 (4.5 Hz), and 0.012 (11 Hz). No correction for multiple comparisons. Lines connecting the data points (denoting individual animals) across the frequencies denote the mean for the locomotor task (blue), and for the joystick task (red). Error bars denote the standard deviation of the mean. Significances are indicated according to *p < 0.05, **p < 0.01. Source data are provided as a Source Data file.

Next, we examined whether fast-changing neuronal activity preceded movement execution (Fig. 4A). Paw velocities provide a general measure of movement magnitude independent of specific types of movements. To allow a comparison of the discretized and typically low-frequency spike trains of the sensorimotor cortex with continuous paw movements, we reconstructed the continuous subthreshold activity with a resolution of 10 ms from the spiking activity^45^ (Fig. 4B). This allows the detection of neuronal activity changes that are faster than those signaled by low-frequency spiking events while simultaneously minimizing the high-frequency transients constituted by each spike. Fast-changing activities typically precede large paw velocities (Fig. 4C). To quantify the relationship between neuronal frequencies and paw velocities, we calculated the Pearson correlation coefficient between the paw velocity and the rectified band-pass-filtered neuronal activity (averaged across neurons), with center frequencies ranging from 0.1 to 11 Hz (Fig. 4D). The correlation was highest at 2.3 Hz for the joystick task and highest at 1.1 Hz for the locomotor task (Fig. 4E). For the joystick task, the correlation reached its maximum at a small negative lag for high frequencies (Fig. 4F), whereas the peak for the locomotor task did not significantly precede the movement for any frequency band. A similar result was achieved without reconstruction for non-sorted neuronal data, for which the thresholded spikes were pooled (Supplementary Fig. 7A–C). Therefore, the peak correlation and lag were consistent with a movement execution function only for high frequencies.

### Frequency-specific decoding of paw movements

**Fig. 5:**
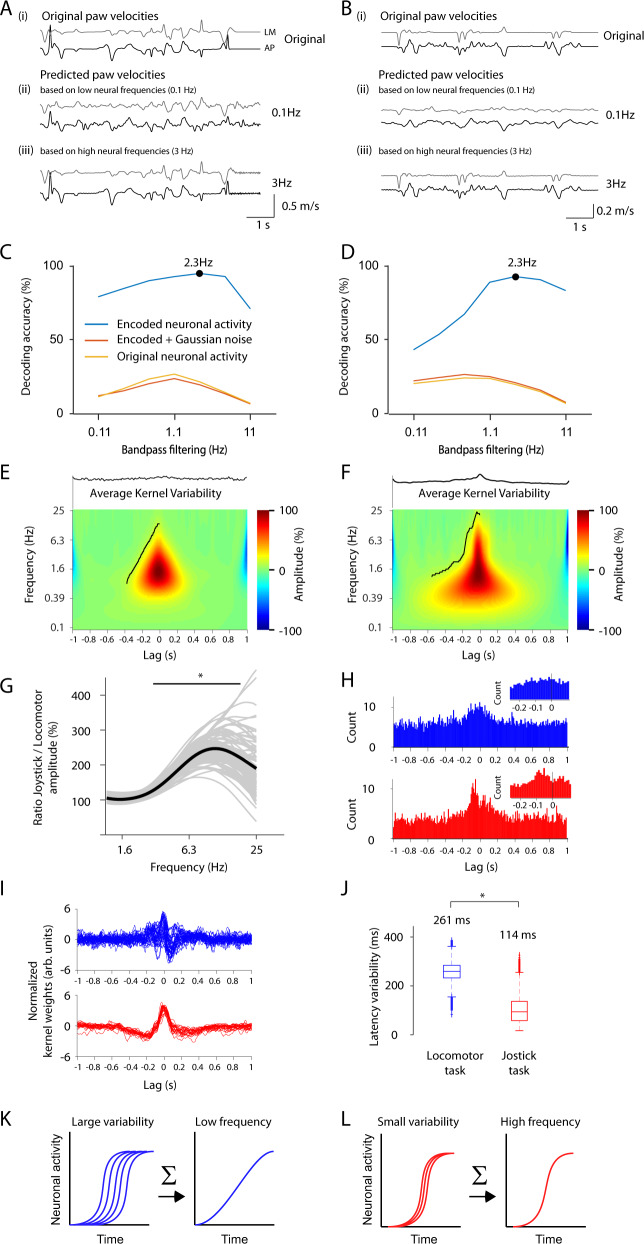
Optimal frequency for decoding behavior. **A** Higher neural frequencies predict paw velocities better than lower frequencies. Original paw velocity in the locomotor task (1). Decoded paw velocity using low (2) and high (3) neural frequencies. The gray curves depict movements in the lateral-medial (LM) direction, black curves refer to movements in the anterior-posterior direction (AP). **B** Indicates the same as panel **A**, but for the joystick task. **C**, **D** Decoding performance is dependent on the neural frequencies for the locomotor task (**C**) and joystick task (**D**) and peaks at 2.3 Hz. To minimize a noise-dependent bias for low frequencies, we decoded the de-noised (encoded) neuronal activity (light-blue line). When we added White Gaussian noise to the de-noised neuronal activity (orange line) this accurately resembled the original neuronal data (yellow line). **E**, **F** Neuronal frequency increases before movement onset. Average wavelet analysis across decoding kernels for all sorted units for the locomotor (**E**) and joystick task (**F**). The amplitude of higher frequencies increases (black line) when approaching the time of movement at lag 0. Top inset: The averaged variability of the decoded kernel across all sorted units measured by bootstrapping across 10-s segments. **G** The amplitude for high frequencies is significantly higher for the joystick task than for the locomotor task. The ratio between the wavelet amplitude of the joystick and the locomotor task is plotted against neuronal frequency. Individual lines are bootstrapped data to show the statistics of the mean. Significances are indicated according to the frequency region for which *p* < 0.05 (*). Corrected for multiple comparisons. **H** The fastest changes in the unit-specific decoding kernel are more focused in time for the joystick task than for the locomotor task. The time point of the largest rectified temporal derivative of the decoding kernels of all sorted units for the locomotor task (blue) and for the joystick task (red). **I** Decoding kernels with a signal-to-noise ratio larger than five. The decoding kernels were normalized to have standard deviations equal to 1. **J** Population activity is more synchronized during the joystick task than during the locomotor task. Latency variability of the full-width half maximum (or minimum) of the unit-specific decoding kernels for the locomotor task (blue) and for the joystick task (red). Two-sided bootstrap corrected for the dependency across sessions (see methods) of the difference of the standard deviation: *p* = 0.011 (Locomotor task: *n* = 57, Joystick task: *n* = 110). The data for each box-plot is bootstrapped and corrected for dependence across sessions (see methods). Box-plots: central mark indicates the median, bottom, and top edges refer to the 25th and 75th percentiles. Significances are indicated according to *p < 0.05. **K**–**L** Illustration of how low temporal jitter across individual neurons may conserve high frequency while a high jitter leads to a low frequency. Source data are provided as a Source Data file.

We subsequently investigated how slowly or quickly changing neural activity could be used to decode paw movements (Fig. 5A, B). For a meaningful comparison, we first had to equalize conditions for slow and fast neural activities. Low-pass filtering, which is needed to extract slow changes in neuronal activity, inherently results in a smoothed neural signal, as well as a higher number of action potentials in the relatively larger time windows required for low-pass filtering. In contrast, smaller time windows are necessary for high-pass filtering, which is applied to extract fast changes in neuronal activity. Smoothing and accounting for more action potentials (i.e., more data) leads to a reduced noise level in low-pass-filtered signals compared to high-pass-filtered signals.

To minimize frequency-specific bias, we conducted decoding on de-noised neuronal activity. We derived the de-noised neuronal activity by predicting the neural activity based on the paw velocity. This inevitably resulted in robust firing rates independent of the frequency. For the de-noising approach, we used the firing rate of each neuron individually and the paw velocity (x and y direction) across the entire session. We collapsed the firing rates and paw velocities by applying a temporal kernel (see Encoding and decoding in Methods). The decoder only had access to two weight parameters for each velocity direction, x and y, to avoid the decoder predicting the correct paw velocity by simply inverting the encoded velocity. The two weight parameters were located at lag 0 and an additional lag defined by the reciprocal of the band-pass frequency. The decoding performance was highest for a frequency of approximately 2.3 Hz (Fig. 5C, D and Supplementary Fig. 8A). By adding Gaussian white noise to the de-noised neuronal activity, we could reproduce the lower optimal decoding band-pass frequency for the raw neuronal data. In sum, this process suggests that (1) de-noised neural activity can be used to investigate the frequency contribution to decoding, and that (2) a frequency of around 2.3 Hz is optimal for decoding paw velocities.

To investigate which neuronal frequency features the decoder used to predict the paw velocity, we applied a wavelet analysis of the unit-specific decoding kernel for the raw spiking neural activity (Fig. 5E, F). The neuronal frequency ramped up from 0.7 Hz (black outline in Fig. 5E, F) 1 s before the movement to 12–24 Hz around 100 ms before the onset of movement. Around 100 ms before the movement, frequencies above 3 Hz had a significantly higher amplitude for the joystick than for the locomotor task (Fig. 5G and Supplementary Fig. 8B–D). As the unit-specific decoding kernel was the result of an average across multiple trials or time points, it is conceivable that the slow component could be the result of the average of multiple temporarily jittered high-frequency components. If this were the case, the variability of the kernel weights would be increased during the low-frequency periods (Supplementary Fig. 9). However, the variability of the kernel weights was not larger during the low-frequency period than during the high-frequency period (see inset Fig. 5E, F). This finding indicates that the low-frequency component was not a result of averaging multiple high-frequency trials but rather was a low-frequency component at the single-trial/time-point level.

As a frequency increase corresponds to an increase in the temporal derivative, we examined the time at which the rectified temporal derivative was the largest for each kernel in each sorted unit (Fig. 5H). In the locomotor task, the time points were less aligned than in the joystick task, resulting in a temporally jittered appearance of the normalized kernel weights (Fig. 5I). To quantify the rise times of the kernel weights, we calculated the time point at which the kernel weights reached 50% of the maxima (or minima, in the case of a negative deflection) (Fig. 5J). The temporal focusing and the higher neuronal frequency in the joystick task may conserve the speed of change of the neural signal when signals from multiple neurons converge on subcortical structures (Fig. 5K–L). Such temporal focusing may ensure that the high-frequency change in the cortical neural activity will be reliably propagated toward the spinal cord.

### Frequency content predicts population state

**Fig. 6:**
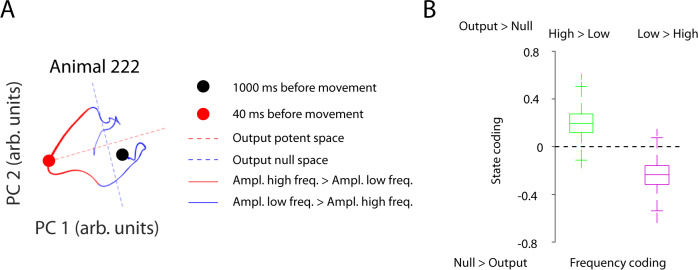
Ratio of high- to low-frequency changes of neuronal data predicts state spaces. **A** Example sessions of dimension-reduced population coding for animal 222. The trajectory is divided into paths for which the high frequency (>1.1 Hz) had a larger amplitude than the low frequency (<1.1 Hz) (red), and into segments for which the high frequency had a smaller amplitude than the low frequency (blue). Output-null (blue) and output-potent spaces (red) are indicated by dashed lines. The thickness of the trajectory indicates the averaged paw velocity. Thicker lines refer to higher velocities. **B** Quantification of the results in panel **A** for all animals. The average state coding was calculated for preferentially high- and low-frequency coding. A negative- or positive-state coding value refers to a dominant null space or output-potent space, respectively. The frequency coding is defined by either a dominance of high or low frequencies of neuronal changes. Dominant high-frequency changes are associated with the output-potent space, whereas dominant low-frequency changes were correlated with the null space. Two-sided paired *t*-test. Data sample dependence correction for the temporal smoothing of 10 samples. High frequency: *p* = 0.033 (*n* = 9439 low-frequency time points), Low frequency: *p* = 0.04 (*n* = 5837 high-frequency time points). The data for each box-plot is bootstrapped and corrected for dependence across sessions (see methods). Box-plots: central mark indicates the median, bottom, and top edges refer to the 25th and 75th percentiles. Source data are provided as a Source Data file.

Previous work has separated planning and motor execution by population code^46^; therefore, we asked whether the frequency code could predict this population coding. The tuning of the paw velocity (Supplementary Fig. 10A, B) was correlated to the change in population code when traversing from motor planning toward motor execution (Supplementary Fig. 10C). As the correlation between the population velocity tuning at planning lags (−1000 to −200 ms) and at execution lags (−40 ms) was minimal, we regarded the population velocity tuning at −40 ms to represent the output-potent space. The space orthogonal to this was regarded as the output-null space. Indeed, the part of the trajectory that corresponded to high-frequency coding was typically associated with high paw velocities and represented the output-potent space. In contrast, the part of the trajectory that corresponded to low-frequency coding was typically characterized by low paw velocities and was located in the output-null space on a single sessions level (Fig. 6A and Supplementary Fig. 10D), as well as in an across-session average (high-frequency coding: *p* = 0.033, *n* = 944, low-frequency coding: *p* = 0.04, *n* = 584, Fig. 6B). Thus, the frequency coding can predict the population code, indicating that frequency coding may be an integral part of movement control, which in turn may represent the underlying biological explanation of the population code theory.

As it is conceivable that slow frequencies related to planning could be governed by the (long-range) input to the population rather than the local activity, we calculated the coherence between the local field potential and the spiking activity for different frequencies during putative planning and execution periods (Supplementary Fig. 11A, B). A planning period was defined as the 2-s-before-trial onset, and an executional period was defined as the 2-s-after-trial onset (see Fig. 3C). The data suggest a decrease in the coherence for low frequencies. The coherence for lower frequency is stronger for the planning period than for the executional period, a relationship that is inverted for the higher frequencies (Supplementary Fig. 11C, D). This supports the hypothesis that the execution is a fast signal that has a local origin and that planning is a slower signal with a more distributed origin.

### High-frequency optogenetic stimulation evokes movements

**Fig. 7:**
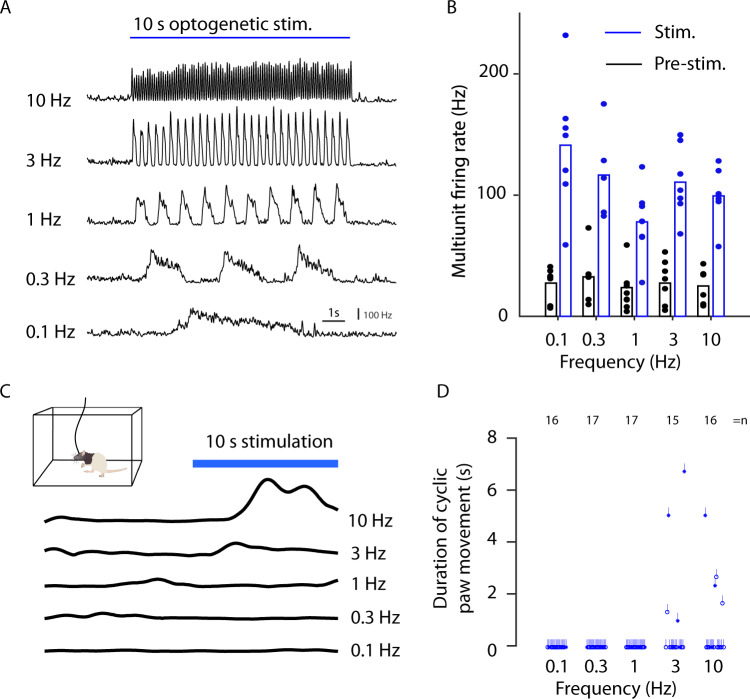
Optogenetic induction of movement. **A** Simultaneous extracellular recordings and optogenetic stimulation at five different frequencies averaged across two animals. The instantaneous firing rate was estimated by threshold crossings in 10-ms-bins for the stimulation period of 10 s. **B** Average firing rate before (black) and during stimulation (blue) for different trials (dots) from two animals. The bars indicate the average firing rate. **C** Optogenetic stimulation in a Plexiglas arena surrounded by seven cameras for behavioral tracking^77^. The behavior was quantified as the amplitude of the velocity for respective stimulation frequencies (black lines). Rat drawing originates from doi.org/10.5281/zenodo.3926015. **D** Behavioral quantification for different stimulation frequencies. Each line corresponds to the duration of repetitive up/down paw movements in a stimulation trial. Optogenetic stimulation did not evoke any cyclic movements in the trials for frequencies 0.1 (16 trials), 0.3 (17 trials), and 1 Hz (17 trials). Only optogenetic stimulation applied with ≥3 Hz induced overt cyclic movements. Source data are provided as a Source Data file.

Next, we optogenetically induced brain activity in the primary motor cortex at different frequencies to examine whether certain frequencies were more likely to evoke paw movements. We tested five different frequencies 0.1, 0.3, 1, 3, and 10 Hz. To minimize the effect of harmonics, the light was varied according to a sinusoidal function. To test whether slow oscillations induced a depolarization block, we measured extracellular activity in close proximity to the optical fiber (Fig. 7A, B). All stimulation frequencies resulted in strong increases in neuronal firing. However, in line with our high-frequency hypothesis, only 3 and 10 Hz resulted in an overt cyclic paw movement (see Fig. 7C, D, Supplementary Movie 1; the average number of behavioral cyclic paw movements for 3 Hz stimulation was 2.3 ± 0.5 Hz, and for 10 Hz stimulation was 3.4 ± 1.4 Hz.) This movement threshold between 1 and 3 Hz aligns with the following: (1) The movement generation was associated with a time constant of 400 ms (Fig. 3J) (corresponding to 2.5 Hz), and (2) the peak in the correlation between paw velocity and neuronal activity occurred above 1.1 Hz (Fig. 4E).

### Nesting of low and high neuronal frequencies

The evidence of a particular function of different frequencies raises the question of whether those frequencies are related. Recently, hierarchically nested neuronal dynamics revealed by phase relationships of slow and fast local field potentials have been proposed to implement such multi-timescale behavioral organization during locomotion^47^. Inspired by these findings, we calculated the cross-frequency coupling of slow 0.1 Hz neural changes with the relatively faster 3 Hz changes (Supplementary Fig. 12). The 3 Hz amplitude was strongest around phase 0, corresponding to the peak of the 0.1 Hz oscillation, indicating a clear cross-frequency coupling between 0.1 and 3 Hz and suggesting a nesting of higher-frequencies in lower-frequencies.

## Discussion

We introduced an alternative approach for investigating the separation of planning and execution of movements. Our approach was inspired by previous work which demonstrated how difficult this separation can be even with tailored task designs. Zimnik et al.^19^ found that the effect of microstimulation in the supplementary motor area (SMA) on the timing of movement initiation, measured as reaction times, depended on the degree of motor preparation. However, a simple model was able to assign a common impact on the probability of movement initiation across all contexts. The SMA has previously been credited with the cognitive processing of motor preparation. However, Zimnik et al.^19^ found that the SMA seems to also be involved in direct initiation in movements. This finding suggests that the classical approach of a go-cued task combined with classical statistical analyses might mask overlapping planning and execution-related neural activity.

Of particular interest for our study is the intermingling of factors, which hampers a clear definition of the contribution of an area. We observed this intermingling of preparatory activity and motor execution between M1 and M2. Previously, a hierarchical relationship between M2 and M1 had been proposed^40–42^. On the one hand, M2 neural activity precedes local M1 activity on a single-trial level^40^, can predict the activity of M1, shows earlier changes during learning^41^, and provides crucial information to M1 for movement accuracy^42^. On the other hand, both M1 (CFA) and M2 (RFA) have been shown to be relevant to planning upcoming movements^4^. We also found indications for planning and execution in both areas but with longer modulation durations in M2 than in M1. These longer modulation durations of M2 support the planning role of this brain area, as planning typically develops over longer time periods. At the same time, some neurons in M2 also showed short modulation durations, suggesting a role in movement execution along the lines of Zimnik et al.^19^.

Our approach offers a way out of this intermingling issue, as the randomized behavior minimizes any behavioral bias. Of course, our approach comes with limitations. The lack of a clear task structure complicated the analyses. Direct comparisons with the existing literature are not always straightforward, as classical statistical tests or more recent advanced approaches typically rely on task phases with multiple identical repetitions. We value these classical approaches because they are typically quite intuitive and have resulted in seminal findings. Thus, we see our randomized movement approach as a complementary method that can provide insights from a different angle but should not replace the existing approaches.

Our study relies on randomized movements, which could be considered as sub-movements. Sub-movements are a long-known phenomenon^48^, typically occurring during the conduct of longer movements that are often investigated in forearm tasks of non-human primates^49–51^. In these experiments, a small sub-movement with a velocity peak occurs every ~ 300 ms. An open debate exists on whether sub-movements rely on extrinsic factors, such as feedback loop delays between the visual system and internal corrections^48,52^, or intrinsic neuronal oscillators working as engine[s] of (sub)-movements^53^.

As we did not train our rats to conduct long movements, the rats were completely free to generate movements according to their inner clock. The feedback from muscles and joints as well as the skin might also be interpreted as external factors, but their impact would be rather on the sensory side and thus should be considered a consequence of the movement. The self-paced movements resulted in an average peak-to-peak velocity time of 210 ms in the locomotor task and 230 ms for the joystick task. We consider this to be sufficiently close to the range of reported sub-movement durations of 1–4 per second to assume that we are dealing with the same mechanism. The slightly shorter sub-movement durations might be related to species differences, as our study was conducted on rats whereas the majority of previous studies were on humans and non-human primates. We found a correlation to the sub-movement duration on a neuronal level: The shortest neuronal modulation durations were in the 300-ms range (locomotor task: 0.36 ± 0.09s, joystick task: 0.27 ± 0.06s; see Fig. 1). However, most of the modulation durations were longer (longest durations in the locomotor task: 1.6 ± 0.37s, joystick task: 1.2 ± 0.37s).

A question remains as to how those longer modulation durations relate to the short sub-movement durations. Here, our optogenetic results provided direct evidence for a low-pass filtering effect: Both 3 and 10 Hz stimulation resulted in 2.3–3.4-Hz movements. This filtering effect explains our findings as well as previously described time scales of movement generation in the 3-Hz range. Reversing the argument, fast-changing population activity can be extracted by a classic high-pass filter. Fast changes refer to, for instance, changes from a high firing rate to a low firing rate or vice versa. Adaptation mechanisms^54–58^ at any stage between the cortex and the muscles could serve as the biological equivalent of such a high-pass filter (see Supplementary Note 2). In this study, we focused on a relatively low-frequency spectrum of neural changes. We found a breaking point around 1.1 Hz and used this to divide the frequencies in a lower range referring to planning, and in relation to this, in a higher range contributing to the execution of movements.

Planning or preparatory activity can refer to individual movements as well as movement sequences. As we performed the analysis over the entire session, there might have been a bias toward preparatory activity for individual chunks of movements (or sub-movements), as only occasionally occurring sequences might be averaged out. The break point at 1.1 Hz lies within the range of 0.3–6 Hz oscillations of local field potentials (LFPs), which have been considered to be low frequency^51,59,60^. In line with our study, these lower frequencies have been hypothesized to be relevant for movement conduction. In particular, LFPs in the range of 3–6 Hz have been reported to be time-locked to sub-movements^60^, and at 3 Hz in particular after a skill had been learned^51^. As LFPs have a clear relationship with the neuronal population as well as individual unit activity, and because of the similarities to previously reported sub-movement durations of 300 ms, it is conceivable that our findings are related to these previous results. In contrast to Hall et al.^51^, we also observed a low-frequency effect in a task that did not require learning (i.e., the locomotor task). Thus, the slow oscillations seem to be an intrinsically omnipresent phenomenon during limb movements and even during sleep and anesthesia^51^; it also seems that they can grow stronger with training^60^.

Going even lower in the frequency spectrum, LFPs as low as 0.3–2 Hz contain relevant information for decoding^59^. Theoretically, the higher decoding power in lower frequencies could be explained by trivial statistics (see “Frequency-specific decoding of paw movements” in Results). Owing to the lower noise level, low frequencies could be particularly meaningful for conveying reliable information, which would also be reflected in a higher decoding accuracy. Using low frequencies would thus be a productive approach to biology. However, our results provide evidence that neuronal changes of around 2.3 Hz were best suited for decoding purposes after our de-noising strategy, which removes this bias. This finding indicates that part of the strong decoding power of the very low frequencies (<2.3) was due to statistics, while the previously reported link between sub-movements and 3 Hz^51,60^ might explain the good performance in this slightly higher frequency range. Our results confirm the significance of the frequency range around 3 Hz, emphasizing that their relevance is not simply explained by statistics. Bansal et al.^59^ also argued that the low-frequency signal is an event-related potential and not an oscillation intrinsic to these cortical areas. The authors assumed that the oscillatory nature of the signal observed in classical behavioral tasks arises from the repeated nature of the movements^61^. By design, our task only contains minimally repetitive elements and yet shows a strong correlation with the low-frequency spectrum of neuronal changes. Thus, our results argue against an explanation of slow frequency changes solely based on repetitive movements.

The concept of different time scales for controlling behavior is widely applied across species including *Caenorhabditis elegans*^47^, Drosophila^61^, zebrafish^62^, zebra finch^63^, and mice^64^. Recently, hierarchically nested neuronal dynamics revealed by phase relationships of slow and fast local field potentials, have been reported to implement such multi-timescale behavioral organizations during locomotion^47^. Inspired by this approach, we showed that 3-Hz changes have a slightly larger amplitude around the maximal activation during 0.1 Hz oscillation. This coupling is a sign of nesting, demonstrating the embedding of faster oscillations into slower ones. This result provided further evidence for the assumption that such hierarchically nested activity patterns may be a common mechanism for organizing and regulating neuronal dynamics across time scales^47^.

The frequency-based separation of motor planning and execution proposed here can be integrated into conceptual frameworks of motor control. According to the concept of dynamic systems, for example, the null-space theory^46^, a frequency-based separation of motor planning and execution would allow both processes to work in parallel. Thus far, the null-space theory has been tested with trial structures with temporally separated planning and execution periods^65^ or with sensory-driven motor execution^66^. For intrinsically planned continuous movements, our results suggest that two independent population state spaces can be generated in the frequency domain, one based on high and one on low frequencies. The concept of separate neuronal populations for motor execution and motor planning (e.g., by projection- or genetically defined neurons^67,68^) assumes a complete separation of the signals. However, genetically defined spinal-cord projecting neurons have been shown to encode not only motor execution but also motor planning^68,69^. Our proposed high-pass filtering mechanism could be a way to expose the motor-execution component by decreasing the planning component. This could explain why the fastest change of the spinal-cord-projecting neurons occurred after the go cue, and why this change was faster than that of the thalamic-projecting neurons^68^. Therefore, the separation of the processes by means of slow and fast dynamics could facilitate simultaneous parallel motor planning and execution within the same neuron, be it in the conceptual framework of dynamical systems or based on identified neuronal subtypes.

The separation of motor planning and execution by means of different frequencies of neuronal activity requires a relatively slowly evolving motor planning. This prerequisite makes intuitive sense, as planning and decision-making rely on accumulating internal or external evidence^70–72^. Thus, motor planning-related neuronal activity changes slowly and hence can be stopped from percolating to the muscles by a high-pass filtering mechanism based on neuronal adaptation. Thus, our proposed mechanisms are able to simply explain the simultaneous implementation of intrinsic motor planning and execution.

## Methods

### Animals

All animal procedures were in accordance with the guideline RL 2010 63 EU and were approved by the Regierungspräsidium Freiburg. In this study, we used six male Long–Evans rats (400 g, Janvier), which were implanted at the age of 8 weeks and recorded up to 4 months after the implantation. Three to four animals were pair-housed in type 4 cages (1500U, IVC typ4, Tecniplast, Hohenpeißenberg, Germany) before implantation and the animals were singly housed after the implantation in type 3 cages (1291H, IVC typ4, Tecniplast, Hohenpeißenberg, Germany) under a 12 h light-dark cycle (dark period from 8 a.m. to 8 p.m., time span of training and experiments). Prior to the initial behavioral training, no behavioral tests were conducted, no drugs were applied, and food (standard lab chow) and water were provided ad libitum. During the course of the experiment, the animals were maintained with free access to food, but the water supply was restricted. Rats were kept at >80% body weight as measured prior to water restriction. For two days per week, free access to water was ensured.

### Animal surgery

Animals were initially anesthetized with isoflurane inhalation followed by intraperitoneal injection of 75 mg/kg Ketamine (MEDISTAR, Holzwickede, Germany) and 50 μg/kg Medetomidine (Orion Pharma, Espoo, Finland). The animals were then put into a transportation container covered with an opaque cloth to facilitate the anesthesia. Once the animals were anesthetized, they were positioned in a stereotaxic frame (David Kopf Instruments, Tujunga, CA, USA) and their body temperature was kept at 36 °C using a rectal thermometer and a heated blanket (FHC, Bowdoin, USA). The anesthesia of the animals was maintained with approximately 2% isoflurane and 0.5 l/min O_2_. For pre-surgery analgesia, we subcutaneously (s.c.) administered 0.05 mg/kg Buprenorphine (Selectavet Dr. Otto Fischer GmbH, Weyarn/Holzolling, Germany). Every other hour, the animals received an s.c. injection of 5 ml isotonic saline. Moisturizing ointment was applied to the eyes to prevent them from drying out (Bepanthen, Bayer HealthCare, Leverkusen, Germany). The skin was disinfected with Braunol (B. Braun Melsungen AG, Melsungen, Germany) and Kodan (Schülke, Norderstedt, Germany). To perform the craniotomy, the skin on the head was opened along a 2 cm long incision using a scalpel. The exposed bone was cleaned using a 3% peroxide solution. Self-tapping skull screws (J.I. Morris Company, Southbridge, MA, USA) for reference for extracellular recordings were placed over the cerebellum. Craniotomies were drilled bilaterally extending from −2 to + 5 mm in the anterior-posterior direction and from + 1 to + 4 mm in the lateral-medial direction relative to Bregma. 22 tungsten electrodes (200 to 600 kΩ impedance, polyimide insulation, WHS Sondermetalle, Grünsfeld, Germany) were implanted at a depth of 1.2 mm in each hemisphere. Electrodes were implanted according to the area borders given by the online brain atlas from Matt Gaidica^73^ and CFA and RFA were delineated according to Neafsay and Sievert^30^ and Rouiller et al.^3^ (Fig. 1E). Three rows of six electrodes each, oriented in the medial-lateral direction, were implanted in the anterior-posterior direction. The fourth and last row consisted of four electrodes, oriented in the medial-lateral direction (see Fig. 1E). Occasionally, we had to cut some electrode wires, in order to not destroy blood vessels at the implantation site (e.g., rat 221, left hemisphere, last electrode row). Kwik-Cast (WPI, Sarasota, FL, USA) was used to protect the brain from the dental cement applied in the final step. Prior to this, Mill-Max connectors (Mill-Max, Oyster Bay, USA) from each hemisphere were glued together to form a 4 × 13 pin connection matrix. The last and first four pins were connected to the two skull screws over the cerebellum to serve as reference and ground. Finally, the assembly was fixed using dental cement (Paladur, Kulzer GmbH, Hanau, Germany).

### Behavioral tasks

Animals were encouraged to move with as little repetition as possible. In the locomotor task, two servo motors positioned a waterspout at various locations within an arena of 30 × 40 cm. Every 10 to 30 s, a valve ejected a drop of water that remained in the mesh until the rats consumed it. To prevent the rats from following the movements of the waterspout, we introduced dummy moves: First the waterspout performed a dummy move without giving water. One second later, it moved to a new position where it let out a water drop. The third and last move was again a dummy move. Even for an experienced animal, this procedure resulted in multiple water drops distributed across the mesh at any given time point. The fact that the rats did not collect all water drops indicates that the animals could not predict where the water was let out and had to actively search for it. This task required minimal training, as indicated by the stable paw velocities over all of the sessions. Thus, we used all sessions for data analysis (Supplementary Fig. 1A).

In the joystick task, the animals had to learn to grab a joystick-like manipulator as a first step. The manipulator was based on a manipulandum for rodents^74^. Instead of having to reach out for the joystick, it was placed right below the right front paw. The naïve rats typically explored the arena in which the joystick was placed. As the animals placed the paw by chance on the joystick, the joystick vibrated and a liquid reward was given as long as three requirements were met: (1) The rats had to keep holding the joystick with the right front paw, which we controlled for via force sensors on the joystick. (2) The left front paw had to be placed on a force sensor plate, which was placed to the left of the joystick. (3) The rats’ heads had to cross an infrared sensor. This ensured that the animals had to learn to use their right front paw to manipulate the joystick rather than the left paw or the mouth.

The vibration of the joystick was implemented by clamping the current of the two motors according to two independent Gaussian processes and served two purposes: (1) It made the animals aware of the joystick. (2) The vibration of the joystick increased in amplitude during the course of 10 s (the maximum vibration amplitude resulted in an average acceleration of 1.5 m/s^2^) such that, unless an animal held the joystick firmly, it would lose the grip and thus not receive a reward. Altogether, these measures resulted in automatic training by which the rats learned to hold the joystick during the maximum vibration amplitude within 10 sessions. Once the rats had developed a firm grip on the joystick, the motors were turned off and the rats received a reward when they actively moved the joystick. Moreover, the rats only received rewards when they moved in a direction or to a position that had not been visited recently (see below). The joystick could be moved within an arena of 40 × 40 mm.

The arena was divided into 5 × 5 bins and the direction of movement was divided into eight bins. For each bin, we stored the amount of remaining reward. Whenever the rats visited one bin, the amount of remaining reward, r, in that bin was decreased to r −Δ*r*. The amount of reward that was decreased, Δ*r*, was distributed among all other bins. Thus, if the rats preferred one bin, the reward within that bin disappeared completely after 20 s. It took up to 15 sessions for the animals to start to move the joystick non-repetitively (Supplementary Fig. 1B). Before the rats started to move randomly, they typically tried to pull the joystick only in one direction (typically towards the rat). This resulted in minimal overall movements since the joystick was stopped by the edges of the arena (the 40 × 40 mm arena). Only once they realized that they could move in all different directions did the amount of total movement increase. For data analyses, we used data from sessions 15 to 35.

### Quantifying behavior

We could relate the joystick position and movement to the egocentric coordinates of the rats because they had to take a defined pose in the joystick task. To enable a comparison of the locomotor task and the joystick task, it was necessary to quantify the behavioral variables in a similar way. To achieve egocentric tracking for the locomotor task, we tracked the paws, head, chest, and belly of the animals. Using those coordinates, we aligned the movements of the right front paw to egocentric coordinates. The neck velocity was calculated from the head, chest, and belly coordinates. Those body parts were tracked by painting them in distinct colors. The head of the rat did not have to be painted because of the black hood of Long–Evans rats. To ensure that all body parts could be tracked, the cameras were placed below the arena. Two to four cameras (Stingray, F033C IRF CSM, Allied Vision Technologies) were used in the locomotor task. The noise of the tracking was estimated to be 0.79 cm/s (estimated when the paw was standing still on the mesh) and was subtracted from the paw velocity estimates.

### Data acquisition and preprocessing of extracellular recordings

Extracellular signals were band-pass-filtered, amplified, and digitized using the INTAN (Intan Technologies, Los Angeles, California) head stage attached to the Mill-Max matrix connector at the animals’ heads. To maximize comfort for the animals, we stripped the ultrathin INTAN cable and suspended it with a 1.5-m long ultralight spring with a 1.5 mm diameter. The long recording cable allowed the rats to move between the locomotor task and the joystick task without having to be disconnected and reconnected. The rats either were in the joystick arena for the entire session or began with 30 min on the locomotor task, after which a door was opened allowing them to walk into the joystick arena for 40 to 90 min. In the case of a dual-task session, we always began with the locomotor task because the color markers used for the locomotor tracking faded over time.

The extracellular recordings were sampled at 30 kHz and were de-noised offline. First, 50 Hz and the corresponding harmonics were removed using a 20-ms template estimation. The activity across all channels was demeaned using a median filter. Spike sorting was conducted on high-pass-filtered data with a cutoff frequency of 300 Hz. Spike snippets were extracted from peak aligned events that crossed a threshold of four times the standard deviation. Only spikes with a negative peak were considered. The spike window was −0.5 to 2 ms around the peak amplitude (resulting in 76 values for each spike). To minimize the risk of a sorted unit being a combination of multiple neurons, we applied a conservative threshold for the cluster size. To this end, we used a cluster size that was dictated by the noise level 0.5 ms before the minimum of the spike. Given the typical refractory period of neurons, this noise estimate excluded variability caused by this unit and was therefore a direct measure of the cluster size of this particular unit. Since our electrodes typically had spacing between 300 and 1000 µm, we sorted each electrode separately.

The spikes were sorted in the raw 76-dimensional space without dimensional reduction. For each sorted unit, the spike sorting algorithm had two phases. First, the algorithm estimated a suitable seed spike. Second, the corresponding waveform was optimized iteratively until the spike assignments of that unit remained constant. The clustering algorithm selected a seed spike by calculating the average noise level across all units. Afterward, the algorithm randomly chose one spike and counted the number of neighboring spikes within this average noise level. Those spikes were called the spike-neighborhood. This procedure was repeated for 500 randomly chosen spikes to maximize the chance of finding a globally optimal seed spike. The spike that had the most neighbors was selected as the seed for a unit. To optimize this spike seed, the noise level for the neighboring spikes was recalculated, the new neighborhood was calculated given this new noise level, and the new average waveform was calculated. This procedure was repeated until the neighborhood remained constant. The spikes within the noise-defined neighborhood were considered to belong to one sorted unit. For this unit, the spike sorting was finished at this point, and it was not considered for further spike sorting. For the remaining spikes, the algorithm restarted phases one and two to search for the next sorted unit. This procedure was stopped when it resulted in sorted units with spike rates lower than 0.1 Hz.

We regarded a unit as a single unit when the number of spikes within an interspike interval of <2 ms corresponded to a smaller firing rate than the average firing rate of the unit. To define the degree of decorrelation across neurons, we used the μ-rate^45^, which is the minimum spike rate in the spike-triggered spike average between two neurons (cross correlogram). The cross correlogram was calculated over a period of −10 to 10 s with a 10 ms binning. We did not calculate the μ-rate from a neuron to itself since that would reflect intra-neuronal processing (adaptation and refractory period) rather than the decorrelation of the population. The μ-rate corresponds to the average spike rate if the spikes of the two neurons occur independently of each other; the μ-rate would be 0 in the case of a lag with no corresponding spike pairs. The μ-rate percentage was calculated by dividing the μ-rate by the average firing rate.

### Single and multiunit velocity modulation

As a general way to relate behavior to neural activity on a single unit or multiunit level, we used a generalized form of a spike-triggered average of the paw velocity, which we denote as activity-weighted distribution (AWD). First, instead of taking discrete spikes, we weighted the behavioral variable (paw velocity or position) with continuous neuronal activity. This continuous activity was the instantaneous firing rate smoothed with a Gaussian kernel with a standard deviation of 50 ms. Second, instead of averaging the behavioral variable, we calculated the distribution for the behavioral variable. A distribution was formed by binning the complete velocity range into 10 equally sized bins. Each bin quantified the average activity across the velocity range of the corresponding bin (see Supplementary Fig. 2). In contrast to the linear average in the classical spike-triggered average, the distribution of the behavioral variable allowed us to consider nonlinearities, e.g., exponentially increasing firing rates with linearly increasing velocity.

According to a traditional spike-triggered average, the relationship between neuronal activity and behavior was calculated at different temporal lags between neural activity and behavior. In this work, we used lags between −4 and 4 s with a temporal resolution of 10 ms. For large delays beyond 3 s, the neuron was typically no longer modulated by behavior. We used the average activity between 3 and 4 s to calculate a baseline activity. This baseline activity was subtracted from the AWD. The average velocity modulation at each lag was calculated by taking the mean of the absolute value of the subtracted AWD (Fig. 1F, G). The duration and the lag of the modulation were calculated by first extracting the peak modulation. We then traced this modulation backward and forward in time until the modulation was <80% of the peak modulation. The temporal difference between those two time points was defined as the duration of the modulation (Figs. 1H, I, 2B, C, and D). The average between those time points was denoted as the temporal lag of the modulation. We took the average time of the 80% start and stop time, since this resulted in a more accurate estimation than the peak time. This was due to the frequent occurrence of plateaus in the velocity modulation. During these plateaus, a small fluctuation of the neuronal signal within the noise level can make the peak appear at any time point along the plateau.

To determine whether a unit was modulated by velocity, we calculated the mean and standard deviation of the velocity modulation at the two extreme lags of the normalized velocity modulation (−4 to −3 s and 3 to 4 s). This mean was subtracted from the velocity modulation and the result was divided by the standard deviation to calculate the normalized velocity modulation. A unit was regarded as modulated if this velocity modulation was larger than 10 (arbitrary units). The variability of the velocity modulation was calculated by dividing the firing rate variance by the average firing rate in each bin of the lookup table that is used to calculate the velocity modulation (see Supplementary Fig. 2). The normalized variability for each sorted unit was calculated by dividing the variability of the velocity modulation by the variability at the baseline interval (−4 to −3 s and 3 to 4 s).

### Bootstrapping velocity modulation

To estimate the variability of the modulation duration, we conducted a bootstrap analysis (Fig. 1H, I). It would be computationally inefficient to sample all 10 ms bins with replacement. Moreover, two neighboring 10 ms bins were not independent; therefore, we chose to divide each session into 100 segments of equal size and calculate the AWD for each such segment. This resulted in segments that were at least 10 s long, allowing computationally effective bootstrap sampling. We sampled the corresponding 100 AWDs with replacement and calculated the resulting velocity modulation. This procedure was repeated 100 times. For each repetition, we calculated the modulation duration. After this, we calculated the standard deviation across those repetitions.

### Population correlation analysis and trial definition

A population correlation analysis was performed on normalized neural activity. For each unit, we divided the spike trains into 10 ms bins, subtracted the average firing rate, and divided each bin by the standard deviation of the binned activity. This normalized data was organized into a matrix with as many rows as there were units and as many columns as there were time bins. To prepare the data for the correlation, we normalized each column to have an average of 0 and a Cartesian norm of 1 (unit length). Finally, we removed a global population activity that could otherwise bias the correlation analysis.

During short periods (between 500 ms to 10 s), the animals sometimes suddenly froze (both in the joystick and the locomotor task), which resulted in a correlated population activity across the joystick and the locomotor task (average *R* = 0.5). As the activity was correlated across two fundamentally different tasks, it was more likely to reflect a global state change rather than a planning process, which in turn could bias the population correlation. We therefore minimized the contribution of this freezing-related population activity, p, by correlating the population activity at each time bin, *a*_*t*_, with the population activity, and subtracting the population activity according to this correlation: *a*_*t*_ – *p*(*a*_t_*p), where * is the scalar product.

With this normalized activity, we calculated the scalar product (Pearson correlation coefficient) between two population vectors at two different time points (Fig. 3C, D). We only correlated population vectors within a trial. As our behavioral data were not separated into defined trials, we constructed trials using the paw velocity. First, we filtered the paw velocity with a Gaussian kernel of 2 s full-width half maximum (FWHM). To find trials for which a period of low behavioral activity was followed by a period of high behavioral activity, we divided each time point in the filtered velocity by each time point in the filtered velocity 2 s earlier. If this ratio was larger than 2 and a local maximum across time, we regarded it as the central time point of a trial. A trial was then defined as 8 s before and 8 s after this maximum. This classification resulted in 1601 bins of 10 ms in one trial. The correlation was calculated between all 1601 × 1601 pairs of time points within a trial. Finally, as the population vector at one reference time point was correlated with the population vector at all other time points, the correlation would decay with increasing distances from the reference time point. This decay was fitted by an exponential function using nonlinear optimization with a Gaussian cost function (Fig. 3E, F, G, and H). The population correlation decay is a measure of the frequency characteristics of the population dynamics: The reciprocal of this time constant defines the frequency at which a low-pass filter with that time constant attenuates the amplitude to 16% of the original amplitude.

To estimate the behavioral frequency at each time point, we calculated the maximum behavioral frequency (within a window inversely proportional to the frequency) that was required for describing the behavior within the error bounds of the tracking.

### Behavioral impact on population correlation

To test how well the neurons encoded for position (Supplementary Fig. 2B), we divided the egocentric x and y movement coordinates of the right paw into five equally sized bins between the minimum and maximum position value. This resulted in a 5 × 5 element matrix. For each element in the matrix, we calculated the average firing rate of the neuron when the paw was in the corresponding position within ± 50 ms. We used this matrix as a lookup table to estimate the instantaneous firing rate at each 100-ms time bin, given the position at the corresponding time bin. The resulting time course of the firing rate was correlated to the time course of the true instantaneous firing rate binned in 100-ms bins. The same analysis sequence was conducted for x and y velocities.

### Subthreshold reconstruction

We employed the subthreshold reconstruction algorithm SubLab^45^. To summarize, the algorithm uses the spikes of one unit (target unit) to reconstruct its subthreshold activity by means of the spiking activity of the remaining units (input units). The algorithm differs from recent auto-encoders and dimension reduction techniques in three aspects: (1) It does not assume an even distribution of spikes in time (Poissonian or Gaussian models); (2) (subthreshold) activity is not modified, as long as it does not cross the threshold; (3) the algorithm reconstructs the subthreshold activity individually per neuron and, therefore, does not impose any relationship between units. Here we used 10 training epochs and we ran the reconstruction on complete sessions.

We also tested the latent factor analysis via dynamical systems (LFADS) auto-encoder algorithm because it does not require a trial structure and can fit complex dynamics to spiking data^75^. For our data, LFADS smoothed the spike trains in a piecewise continuous way. We observed gaps in the smoothed spike trains. We suspect that these gaps were due to the spontaneous and complex behaviors, which in turn caused the internal states to be reset frequently.

The reconstructed activity was filtered in the following way (Fig. 4C, D, E, and F). High-pass filtering: First, the reconstructed signal was smoothed with a Gaussian kernel with a standard deviation (σ) of 0.14 s. Using the cutoff frequency formula for Gaussian filtering (2πσ)^−1^, this corresponds to a cutoff frequency of 1.1 Hz. Second, we subtracted this smoothed signal from the original reconstructed signal. Band-pass filtering: First, the reconstructed signal was smoothed with a Gaussian kernel with standard deviations of 0.057, 0.14, 0.28, 0.57, 1.4, 2.8, and 5.7 s (2.8, 1.1, 0.57, 0.28, 0.057, and 0.028 Hz), respectively. Second, we subtracted this smoothed signal from the original reconstructed signal. Third, the resulting signal was smoothed with a Gaussian kernel with standard deviations of 0.014, 0.035, 0.071, 0.14, 0.35, 0.71, and 1.4 s (11, 4.5, 2.2, 1.1, 0.45, 0.22, and 0.11 Hz), respectively. Low-pass filtering: The band-pass-filtered signal that was filtered with a low-pass kernel of 0.71 s (0.22 Hz) and high-pass kernel of 2.8 s (0.057 Hz) was referred to as the low-pass-filtered signal. The additional high-pass filtering minimizes the influence from strong low-frequency components. Finally, to get the energy of the filtered signal, we calculated the absolute value of the high-pass-filtered signal.

### Relating population and frequency coding

Output-null and output-potent coding has traditionally been studied during the planning and execution phase of instructed delay tasks. Our behavioral setting does not include a typical trial structure; thus, we defined the planning and execution phase in terms of the lag between the paw velocity and the neuronal activity. To this end, the anterior-posterior paw velocity was multiplied with the neuronal activity for a sorted unit in a bin-wise manner for a given lag and averaged across all bins. Thus, for a given lag, this approach will quantify how each neuron codes for the movement in a linear manner. We used temporal lags from −1 s to 1 s with 10-ms bins. The result is an *N* × 201-dimensional matrix for each session, where *N* is the number of sorted units. Dimension reduction to a 2 × 201 matrix was achieved by taking the largest two principal components. We defined the output-potent space as a one-dimensional space covered by the vector between the origin (0, 0) and the point in the two-dimensional space (spanned by the first two principal components) at a lag of −40 ms. This lag was defined by the notion that executional activity should have a small correlation to the planning activity, which, in turn, translates to the lag at which the correlation to the average activity between −1000 and −200 ms (putative planning activity) was smallest. We choose the upper limit to be −200 ms to minimize the bleeding into executional activity^22,23^. This definition of the lag for the output-potent space maximizes the separation of planning and executional activity, thereby maximizing the chance that the null and potent spaces will be found. Such a biased definition is justified here, since the aim is not to verify the null-space theory but rather to see if it is related to the ratio of high and low frequencies of neuronal changes. The output-null space was orthogonal to this output-potent space. For each lag, we estimated which state the neuronal activity was closest to by means of the difference in magnitude: abs(output-potent) – abs(output-null). If this *state tuning* was positive, we regarded the neuronal activity to be in the output-potent state. If state tuning was negative, we regarded the neuronal activity to be in the null space.

To test whether the frequency coding could predict whether the neuronal activity is in the output-potent or output null space, we assigned a frequency preference for each lag of a certain session. This was done by calculating the difference in magnitude: abs(amplitude of high frequency) –  abs(amplitude of low frequency). A positive *frequency tuning* meant that the neuronal activity had a greater high-frequency component, and if it was negative, the neuronal activity had a larger low-frequency component. Across all sessions, we pooled all lags that had a positive or negative frequency tuning and calculated the resulting average state tuning.

### Behavioral quantification during optogenetic stimulation

For optogenetic stimulation, we used a 200-μm fiber implanted at 1 mm depth in the primary motor cortex of two rats (511 and 512) (AP = 0.5, LM = 2, and DV = 1). The viral vector AAV5 carrying the construct hSyn-hChR2(H134R)-eYFP-WPREpA (UNC vector core, Chapel Hill, NC, USA), was injected at a depth of 1.5 mm with a volume of 1 μl. Each stimulation trial lasted 10 s and the light intensity was sinusoidally modulated according to one of five frequencies: 0.1, 0.3, 1, 3, and 10 Hz with a peak power of 4–12 mW at the fiber tip. As the current that ChR2 can give rise to is smaller for low frequencies than for high frequencies, we compensated with a stronger light intensity for the lower frequencies^76^. Each trial was randomly interleaved with 120 to 240 s.

To quantify the subtle paw movements that result from sinusoidal optogenetic stimulation (Fig. 6C), we first calculated the paw position using FreiPose^77^. For a given trial, we manually selected the camera with the clearest view of the right foot (the optogenetic stimulation was in the left hemisphere). The paw position for each frame was then projected to this camera and a 100 × 100-pixels window was cut out around this projected position. The optical flow was calculated for each pair of neighboring frames (opticalFlowHS object in MATLAB). The paw position for both frames in this pair was taken according to the first frame. The vertical component of the optical flow was extracted because it is the major movement axis during stimulation. Finally, the optical flow was only sampled at pixels with a saturation above 20% (0.2 for saturation of the rgb2hsv function in MATLAB). This saturation threshold was chosen to sample paw movements rather than more unspecific fur movements. Trials in which the rat was grooming, eating, or walking were eliminated from further analysis. The amount of movement for each stimulation frequency was then calculated by averaging the energy in the 0.1, 0.3, 1, 3, and 10 Hz bands (using the spectrogram function in MATLAB with a window size of 100 and overlap of 99).

In addition to the automatic behavioral quantification described in the previous paragraph, in Fig. 6D, we manually quantified how the animal responded to the optogenetic stimulation. To this end, we measured the duration for which the rat performed an abnormal behavior. Abnormal behavior was defined as a paw movement for which the rat was lifting and lowering the right paw towards the original location at least two times. We excluded movements that could be ascribed to walking or grooming, as well as movements that showed coordination between the left and right paw. Although the criteria seemed robust, there was one trial in which rat 512 stretched out the paw abnormally for the 10 Hz stimulation and this was therefore not counted as a cyclic movement.

### Encoding and decoding

We de-noised the neuronal activity data by exchanging the raw neuronal activity with a predicted neuronal activity having a reduced number of parameters. The prediction was based on the paw velocity data. We called this step encoding. The encoded neuronal activity contained considerably less noise because the predicted neuronal activity was the result of a temporal kernel with several orders of magnitude fewer parameters than the original data samples.

To encode the paw velocity, we convolved a temporal kernel with the paw velocity. The temporal kernel had a range of −2 to 2 s with a temporal resolution of 10 ms (sliding window), resulting in a temporal width of *T* = 400. As we used the paw velocity in the *X* and the *Y* direction to predict the neuronal activity for each sorted unit, the temporal kernel was a matrix with *T* × 2 weights (2 × 400 samples). We optimized the weights of the temporal kernel by minimizing the least square error between encoded neuronal activity and the raw neuronal activity. The de-noising eliminated the noise bias that otherwise occurs for lower frequencies. This de-noised—and thereby unbiased—data enabled us to test how different frequencies in the neuronal activity contributed to the decoding of the paw velocity.

Following the encoding, we decoded the paw velocity based on the de-noised neuronal activity. For testing the frequency dependency of the decoding performance, we applied a double sample kernel with the two weights at two different time points relative to behavior: −1/frequency and 0 seconds, respectively. The frequency corresponded to the center frequency of the band-pass-filtered neuronal activity. Thus, the kernel consisted of a matrix with 2 × *U* weights, where *U* corresponds to the number of units. We optimized the weights of the decoding kernel by minimizing the least square error between the decoded paw velocity and the measured paw velocity.

For extracting the unit-specific decoding kernel, we used one temporal kernel (400 samples, as for the encoding) for the *X* and *Y* paw direction. Thus, for the unit-specific decoding kernel, each kernel was a matrix with *T* × 1 weights, where *T* corresponded to the number of weights in the temporal kernel (i.e., 400). The temporal kernel of each unit was optimized independently of the temporal kernel of the other units. We optimized the weights of the temporal kernel by minimizing the least square error between the decoded paw velocity and the measured paw velocity. To investigate which neuronal frequency featured the decoding best, we applied the wavelet analysis of MATLAB (command cwt) with a symmetry parameter of 1.5 and a time-bandwidth product of 2.

### Calculating the phase-dependent amplitude modulation

The neuronal firing rate was band-pass-filtered with 0.1 Hz and 3 Hz, respectively. The instantaneous phase of the 0.1-Hz band-pass-filtered activity was calculated using the Hilbert transform. The phase for all time points was then binned into 16 evenly divided bins. The amplitude of the 3 Hz signal was averaged for those time points that were binned into the same phase bin. To calculate the amplitude of the 3 Hz band-pass-filtered activity, the signal was first rectified and smoothed at 1.5 Hz.

### Statistical procedures

All statistics and graphical illustrations of spiking unit data were corrected for the possibility that the same unit has been recorded during multiple consecutive days (Supplementary Table 3). In the motor cortex, evidence has been provided that tungsten electrodes are able to record the same unit for an average of 3 days, and a considerable amount (11%) of neurons could be recorded for up to 7 days^78^. As we had a recording session almost every day, we conservatively regarded every seventh unit to be an independent data sample. To this end, the degrees of freedom were calculated on the basis of the unit count divided by seven. We made this correction for the *t*-test, the Pearson correlation coefficient, and the ANOVA. For box-plots (using MATLAB’s box-plot function), we plotted the bootstrapped data (using MATLAB’s bootstrap function with 1000 iterations) and adjusted the standard deviation of the bootstrapped data such that it was √7 times that of the original data. In addition, this correction for independent data samples was also applied to a mixed-effect model. The modulation duration difference between cortical areas was also tested using a linear mixed-effect model for which the areas were modeled with an additive random effect and the cortical location was modeled with a fixed effect. All responses from a given electrode were averaged across sessions for a given animal.

For statistical testing, we assumed that the data were normally distributed. The test statistics for the Pearson correlation coefficient, the ANOVA, and unpaired statistics approached a normal distribution for large data samples. For the paired *t*-test, we assumed a normal distribution, as the test distribution was symmetric around zero. Unless otherwise stated, samples were described in terms of mean and standard deviation of the mean.

As we had one less animal in the joystick task (animal 220 lost the implant before it learned the joystick task), all paired tests were done without animal 220 in both the joystick and locomotor task. The non-paired tests were done using all six animals in the locomotor task and all five animals in the joystick task.

### Reporting summary

Further information on research design is available in the Nature Research Reporting Summary linked to this article.

## Supplementary information

Supplementary Information

Description of Additional Supplementary Files

Supplementary Movie 1

Reporting Summary

## Data Availability

For a proper evaluation of all presented results, the entire data is required. As the dataset is quite large with 0.5 Gb per session with more than 100 sessions in total, the data that support the findings of this study are available from the corresponding authors upon reasonable request. Source data are provided with this paper.

## Source data

Source Data

## Code availability

The code for analyzing the data of this study is available from the corresponding authors upon reasonable request. Source data are provided with this paper.
