# Supplementary material for: Distinct dynamics of neuronal activity during concurrent motor planning and execution
Source: Nat Commun. 2021 Sep 10;12:5390. doi: 10.1038/s41467-021-25558-8 (PMC8433382; doi:10.1038/s41467-021-25558-8)
Supplement: Supplementary file 1 — Supplementary Information [file 41467_2021_25558_MOESM1_ESM.docx]

**Supplementary Information**

**
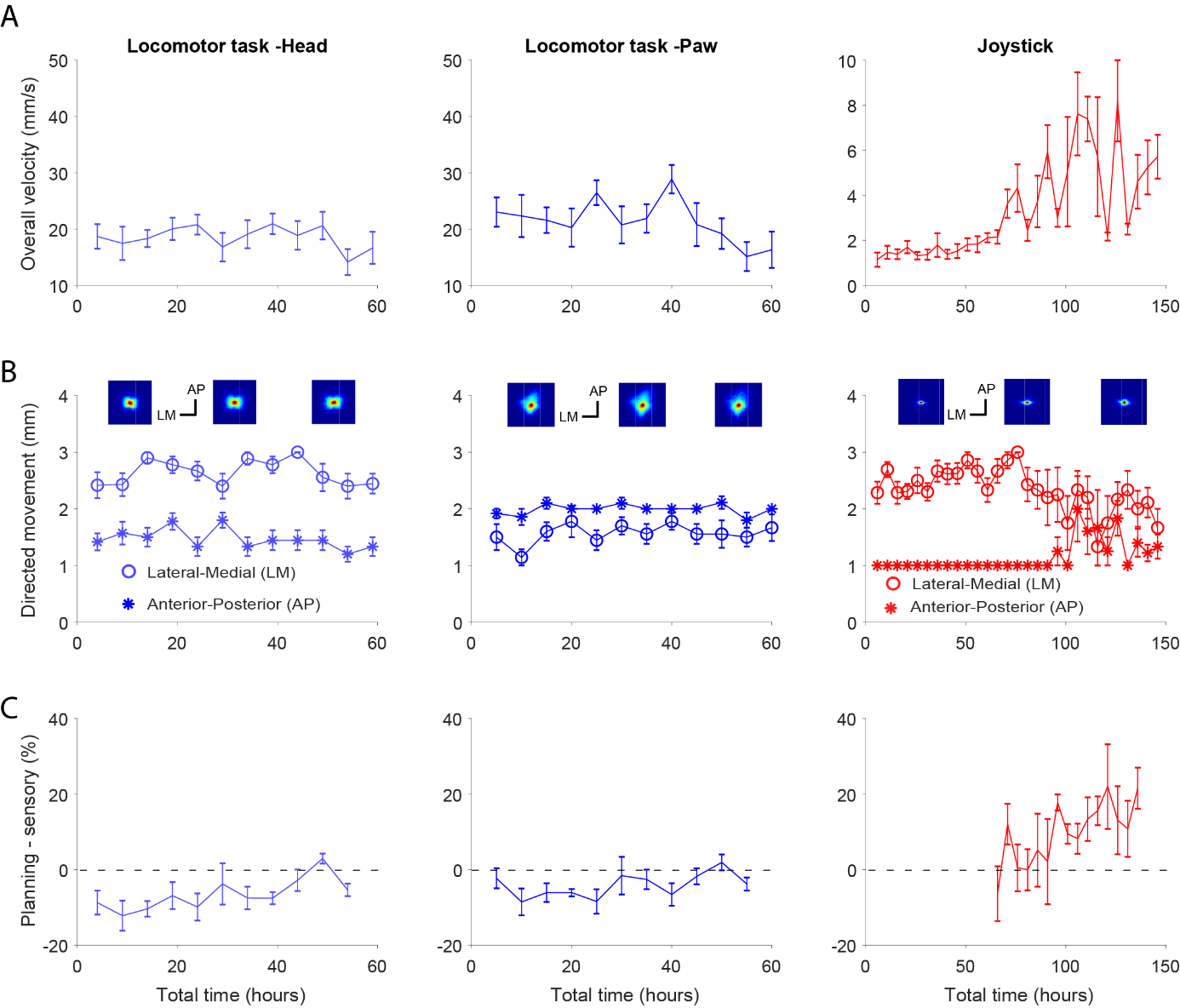
Supplementary Figure 1.** Training metrics. A: The average velocity as a function of the total recording time for the neck movements in the locomotor task (left column), paw movements in the locomotor task (middle column), and paw movements of the joystick task (right column). Error bars denote the standard deviation of the mean. Locomotor task –Head: n=116 sessions. Locomotor –Paw: n=118 sessions. Joystick task: n=213 sessions. B: Spatial transition probabilities in the lateral medial (LM) direction (circles) and in the anterior posterior (AP) direction (stars) as a function of the total recording time according to panel A. The insets shows the transition probabilities at three different time points in a logarithmic scale. Error bars denote the standard deviation of the mean. Locomotor task –Head: n=116 sessions. Locomotor –Paw: n=118 sessions. Joystick task: n=213 sessions. C: The summed velocity modulation for motor planning related neuronal activity (negative lags from -1.1 to -0.1 seconds) minus the summed velocity modulation for sensory integration related neural activity (positive lags from 0 to 1 seconds). Error bars denote the standard deviation of the mean. Locomotor task –Head: n=107 sessions. Locomotor –Paw: n=107 sessions. Joystick task: n=80 sessions. Source data are provided as a Source Data file.


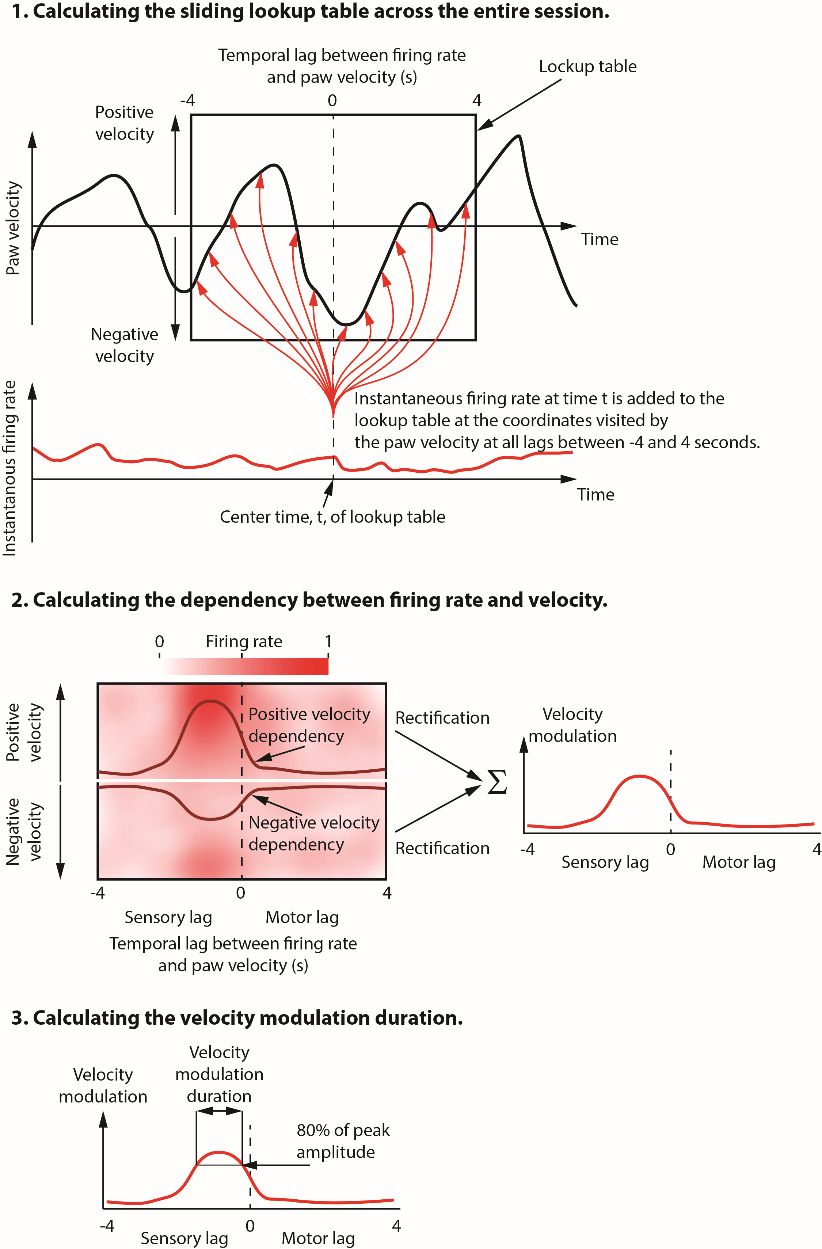


**Supplementary Figure 2.** 1. Calculating the sliding look up table*.* To capture whether the neuronal firing rate depended on the velocity, we estimated the average firing rate for the complete range of available velocities. To relate this information to planning, execution, and sensory processes, we calculated this velocity dependency for different temporal lags between paw velocity and neuronal activity. To this aim, we separated the neuronal signals and the paw velocity data in 10 ms bins. The lockup table was constructed by taking the firing rate value at time t and adding that value to the lockup table for all paw velocities that preceded and succeeded that time point t within a time window of -4 to 4 seconds. Multiple values at one particular coordinate in the lockup table, originating from multiple time points across the session, were averaged together. 2. Calculating the dependency between firing rate and velocity. For large delays beyond 3 s, neurons were typically no longer modulated by behavior. Thus, we used the average activity between 3 and 4 s to calculate a baseline velocity dependency. We subtracted this baseline activity from the lockup table and rectified the resulting values. The average velocity modulation at each lag is the mean of the rectified values. 3. Calculating the velocity modulation duration. The velocity modulation duration is a measure for how long the dependency between firing rate and velocity lasts. The velocity modulation duration is equivalent to the time during which the velocity modulation exceeds 80 % of its maximum.


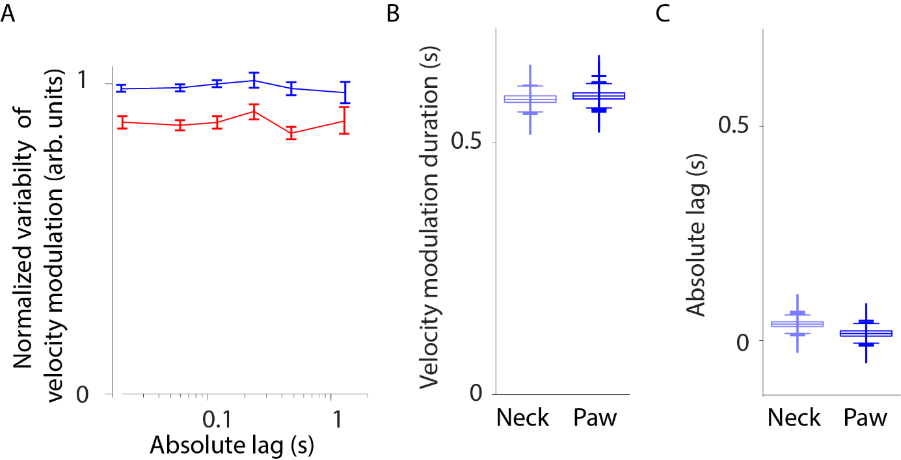


**Supplementary Figure 3.** Longer velocity modulation for larger absolute lags is neither due to a larger temporal scatter, nor due to the neck velocity. A: If the longer velocity modulation duration for larger absolute lags was due to the averaging of a temporally scattered behavioral variable, the variability of the velocity modulation should be higher for larger lags for the locomotor task (blue) and for the joystick task (red). Locomotor task: n=3124. Joystick task: n=1982. Only sorted units with a negative lag (velocity modulation peak) were considered for this analysis, since those units typically had a broader duration for long lags (see Figure 2B). The error bars indicate the standard deviation of the mean. B: Average neck and paw velocity modulation duration. Locomotor task (Neck): n=5547. Locomotor task (Paw): n=5413. The data for each box-plot is bootstrapped to show the statistics of the mean, and corrected for dependence across sessions (see methods). Box-plots: central mark indicates the median, bottom and top edges refer to the 25th and 75th percentiles. The whiskers extend to the most extreme data points not considered outliers, and the outliers are plotted individually using the '+' symbol. C: Average neck and paw velocity modulation absolute lag. Locomotor task (Neck): n=5547. Locomotor task (Paw): n=5413. The data for each box-plot is bootstrapped to show the statistics of the mean, and corrected for dependence across sessions (see methods). Box-plots: central mark indicates the median, bottom and top edges refer to the 25th and 75th percentiles.

**
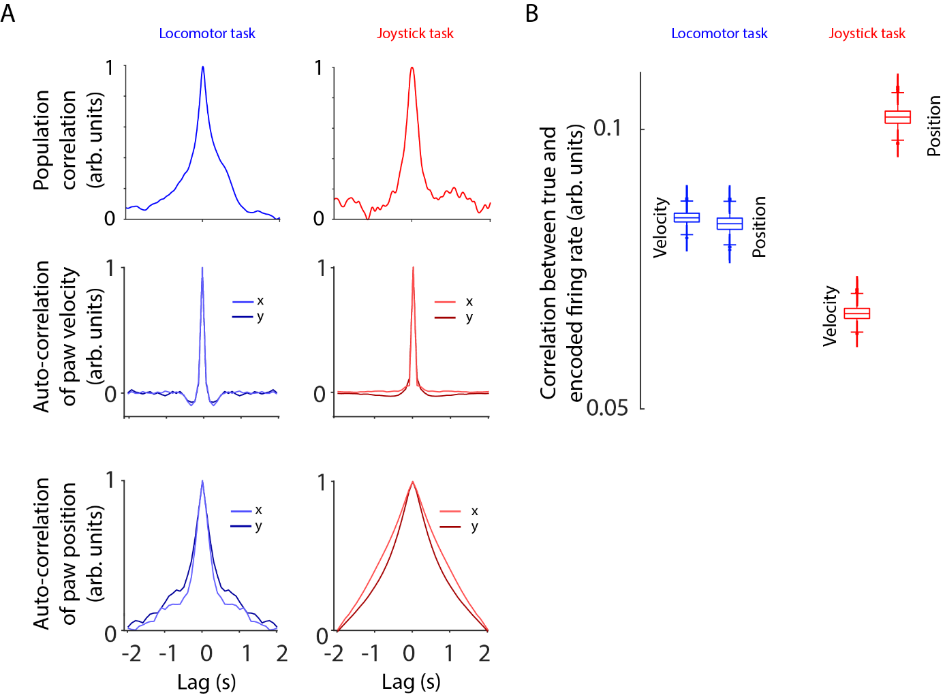
**

**Supplementary Figure 4.** Ruling out a putative behavioral impact on population correlations. A: Population activity correlation for the locomotor task and the joystick task (top row). Autocorrelation of the paw velocity for the locomotor task and the joystick task (second row), and of the paw position for the locomotor task and the joystick task (bottom row). B: Encoding performance using only position or direction of the right paw. Locomotor task (Neck): n=13016. Joystick task (Paw): n=9309. All sorted units were used since encoding performance is a continuum from no- to strong encoding. Box-plots: central mark indicates the median, bottom and top edges refer to the 25th and 75th percentiles. Source data are provided as a Source Data file.


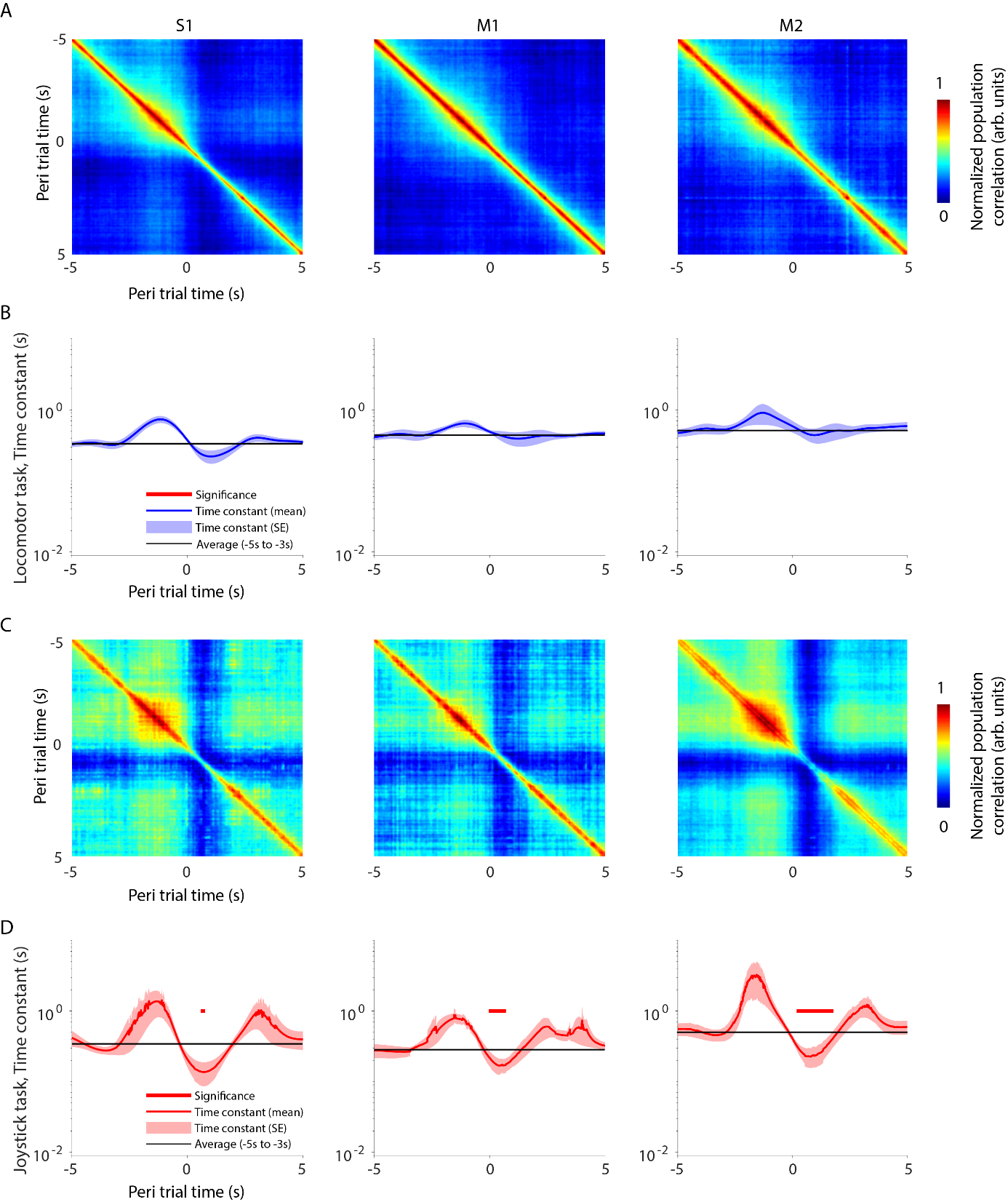


**Supplementary Figure 5.** Population correlation for different areas. A: Population correlation matrix across S1 (first column), M1 (second column), and M2 (third column) for the locomotor task. B: Population correlation time constants (green line) across S1 (first column), M1 (second column), and M2 (third column) for the locomotor task. The pool velocity is plotted as a reference (black line). The shaded area indicates the standard deviation of the mean. C-D: same as panel A -B, but for the joystick task. Source data are provided as a Source Data file.


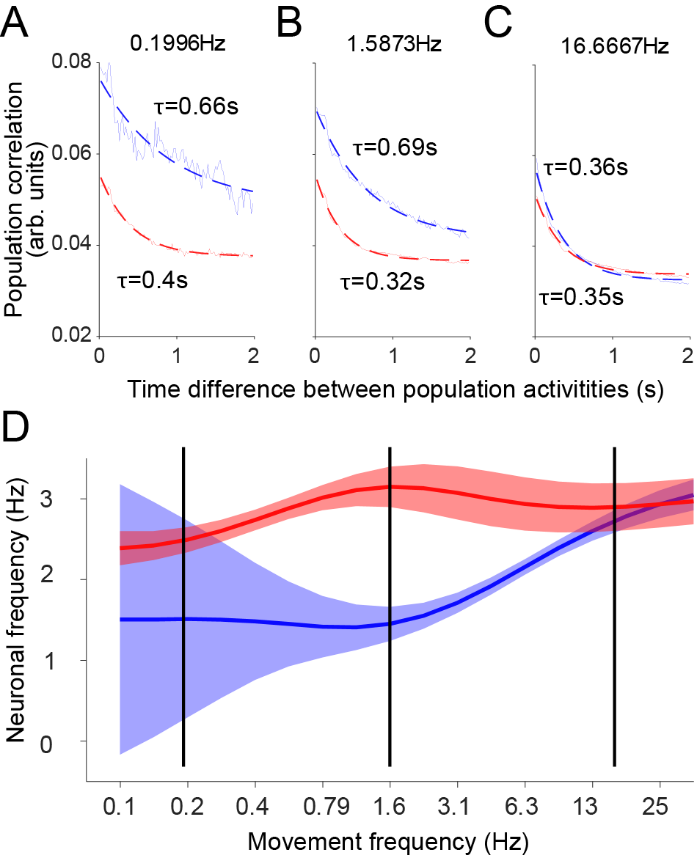


**Supplementary Figure 6**. Neuronal population dynamics for specific movement frequencies. A-C: Neuronal time constants (τ) for three different movement frequencies for the locomotor task (blue) and the joystick task (red) describing how the correlation decayed for increasing time difference between the population activities. The movement frequency is provided on top of each graph with increasing frequency from left to right. D: The neuronal frequency that corresponded to one over the time constant, remained high for all movement frequencies for the joystick task except those below 0.2 Hz (red line). The neuronal frequency increased only for high movement frequencies for the locomotor task (blue line). The shaded area indicates the standard deviation of the mean. Source data are provided as a Source Data file.

**
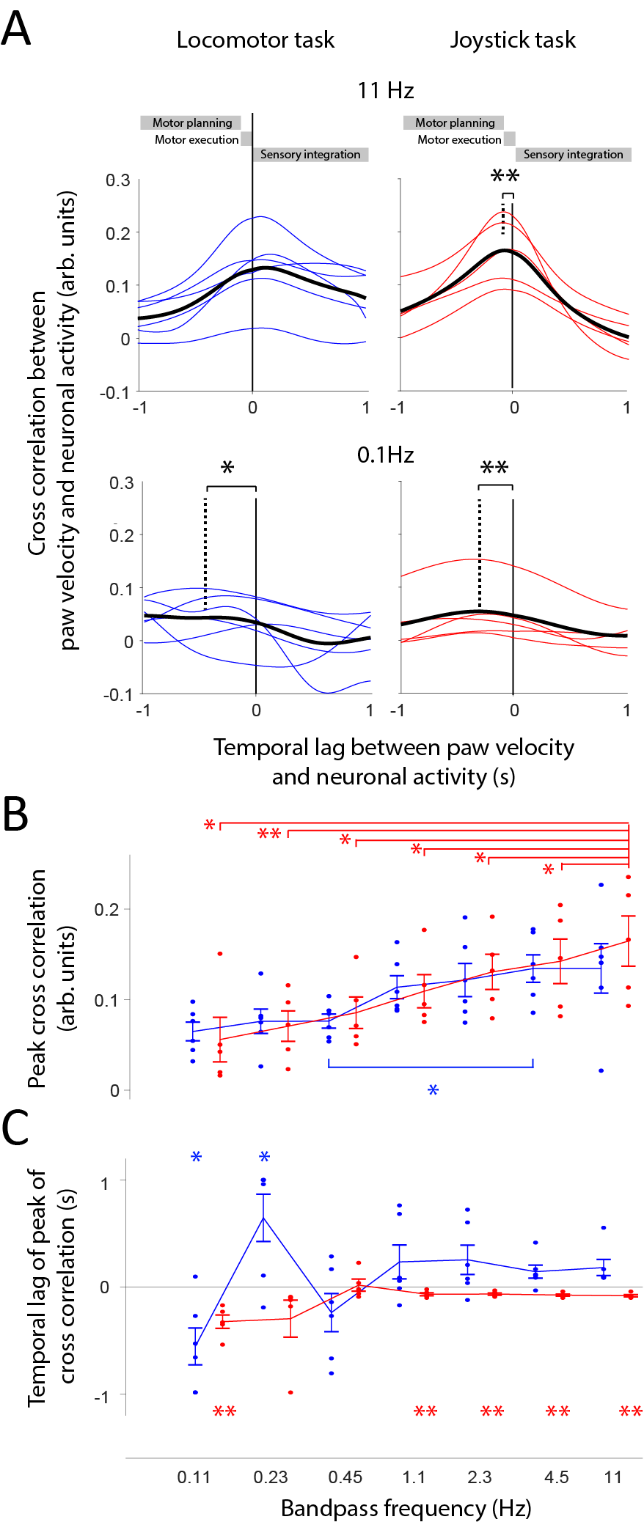
**

**Supplementary Figure 7.** Correlation between paw velocity and the spectral properties of unsorted population activity. A: The Pearson correlation coefficient for different lags between the rectified band-pass (11 Hz) filtered neuronal activity and the paw velocity during the locomotor task (upper-left, two-sided *t*-test, p=0.069, n=6), and the joystick task (upper right, two-sided *t*-test, p=0.001, n=5), and for the rectified band-pass (0.11 Hz) filtered neuronal activity and the behavior during the locomotor task (lower left, two-sided *t*-test, p=0.022, n=6), and the joystick task (lower right, two-sided *t*-test, p=0.0062, n=5). The comparison between cross-correlation values at time point zero and the time point of maximal cross-correlation reveals significant changes with different temporal lags. B: The peak correlation between the paw velocity and the rectified band-pass filtered neuronal activity for seven frequencies for the joystick task (red), and the locomotor task (blue). Two-sided paired T-test. Locomotor task: n=6. p=0.048. Joystick task: n=5. p=0.011, 0.0058, 0.012, 0.035, 0.046, 0.018. No correction for multiple comparisons. Lines connecting the data points (denoting individual animals) across the frequencies denote the mean for the locomotor task (blue), and for the joystick task (red). Error bars denote the standard deviation of the mean. C: Temporal lags of the peak Pearson correlation coefficient (across temporal lags) for rectified band-pass filtered neuronal activity. Two-sided paired T-test. Locomotor task: n=6. p=0.022, p=0.034. Joystick task: n=5. p=0.0062, 0.0056, 0.0024, 0.001, 0.001. No correction for multiple comparisons. Lines connecting the data points (denoting individual animals) across the frequencies denote the mean for the locomotor task (blue), and for the joystick task (red). Error bars denote the standard deviation of the mean. Significances are indicated according to: * p < 0.05, ** p < 0.01. Source data are provided as a Source Data file.

**
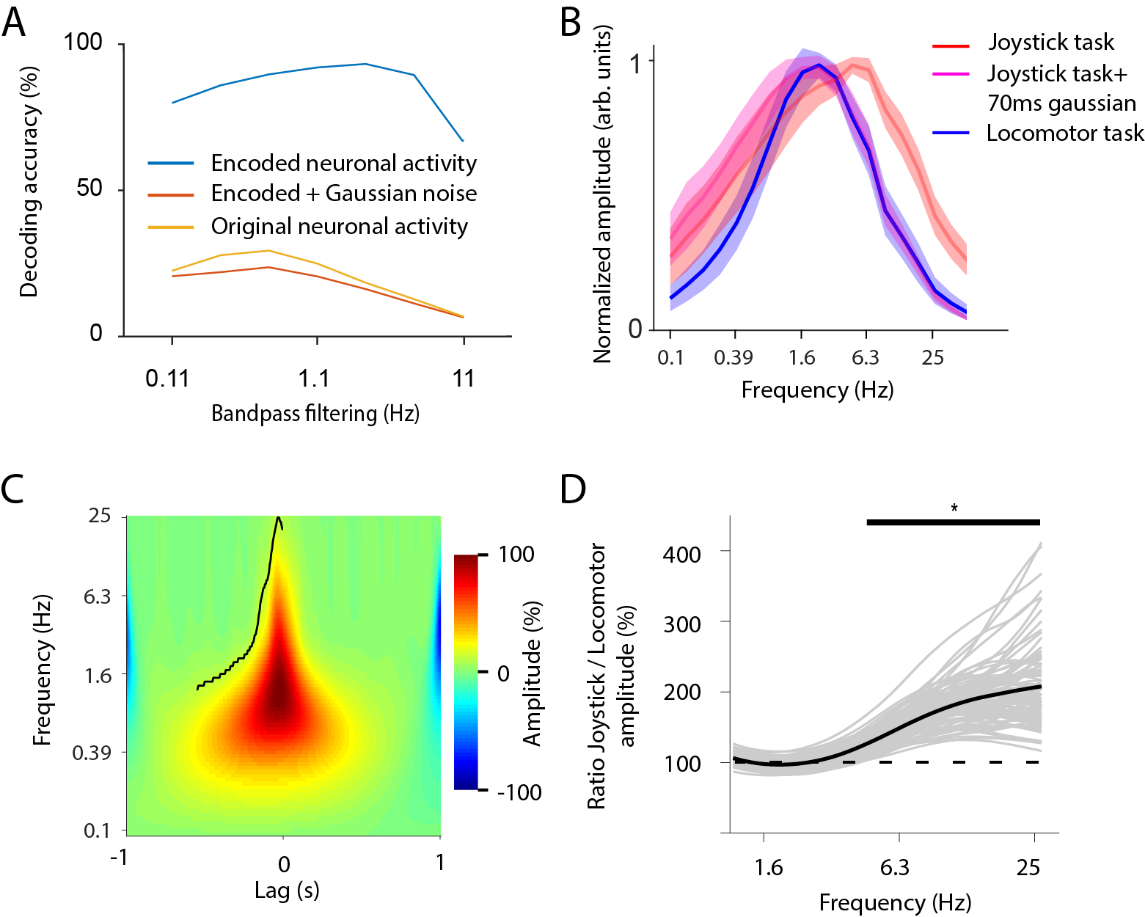
**

**Supplementary Figure 8.** Relation between decoding and behavior. A: Accuracy for decoding neck velocity during locomotion. B: Frequency spectrum of the raw paw velocity in the joystick task (red), in the joystick task when the paw velocity was filtered with a 70 ms Gaussian smoothing kernel (pink), and in the locomotor task (blue). The temporal smoothing of the joystick movements makes the spectrum above 1 Hz indistinguishable from that of the locomotor task. The shaded area indicates the standard deviation of the mean. C: Wavelet analysis of the decoding kernels for the temporally smoothed joystick movements, see panel B. D: The ratio between the spectral amplitude at lag -0.03s for the smoothed joystick movements and the locomotor task visualize that there is a significant difference between the joystick and the locomotor task even when the frequency spectrum of the movement for the two tasks is equalized (see panel B). Individual lines are bootstrapped data. Significances are indicated according to the frequency region for which p < 0.05 (*). Corrected for multiple comparisons. Source data are provided as a Source Data file.


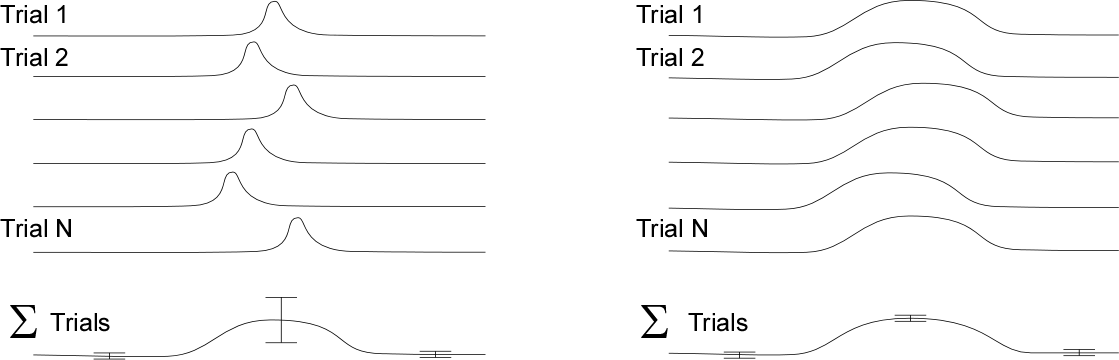


**Supplementary Figure 9.** Illustration of how summation of high frequency components causes large variability. The summation of multiple jittered high frequency components can create a low pass frequency component with large variability (left). The summation of multiple jittered low frequency components can create a low pass frequency component with small variability (right). Thus, we have to rely on the variance in the average to draw a conclusion about the quality of the underlying single trials.


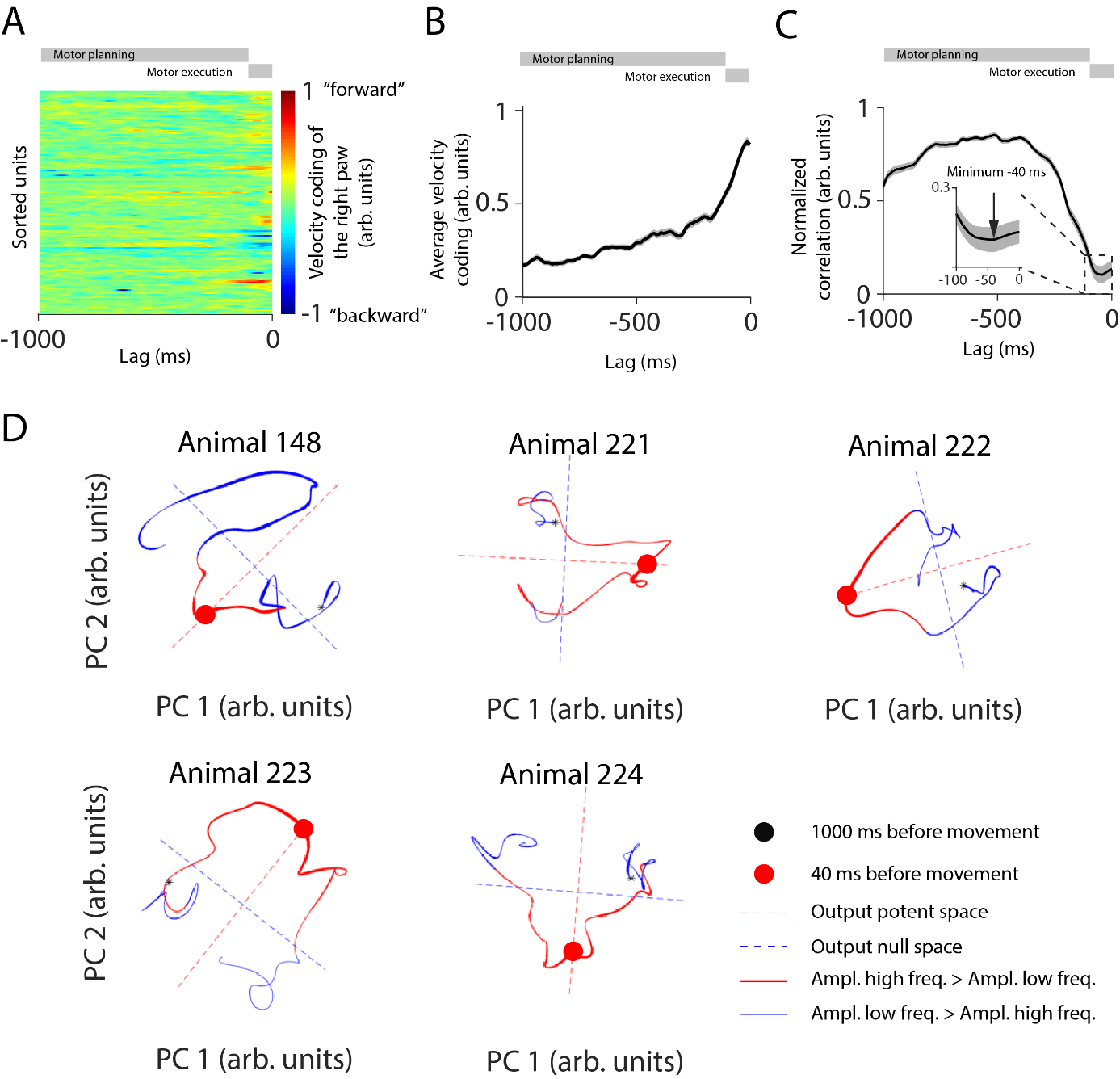


**Supplementary Figure 10**. Ratio of high to low frequency changes of neuronal data predict state spaces. A: Velocity tuning across all units in one session of rat 222 across temporal lags ranging from -1000 to 1000 milliseconds. B: Average velocity tuning across all units, all sessions of all animals. C: Correlation between the average population activity between -1000 and -200 ms and the population activity at all lags between -1000 and 0 ms (x-axis). The correlation reaches a minimum at -40 ms (inset). This minimum describes when the motor execution related activity is maximally different from motor planning related activity and is hence used to define the output potent space. Individual lines are bootstrapped data to show the statistics of the mean. D: Example sessions of dimension reduced population coding for the five animals in the joystick task. The trajectory is divided into paths for which the high frequency (>1.1Hz) had a larger amplitude than the low frequency (<1.1Hz) (red), and into segments for which the high frequency had a smaller amplitude than the low frequency (blue). Output null (blue) and output potent spaces (red) are indicated by dashed lines. The thickness of the trajectory indicates the averaged velocity tuning (B). Thicker lines refer to stronger correlation between paw velocity and neuronal activity. All animals apart rat 148 showed a clear correlation of the output potent space with paw velocities. This rat had the lowest signal to noise ratio and the electrodes covered a smaller cortical area. Source data are provided as a Source Data file.


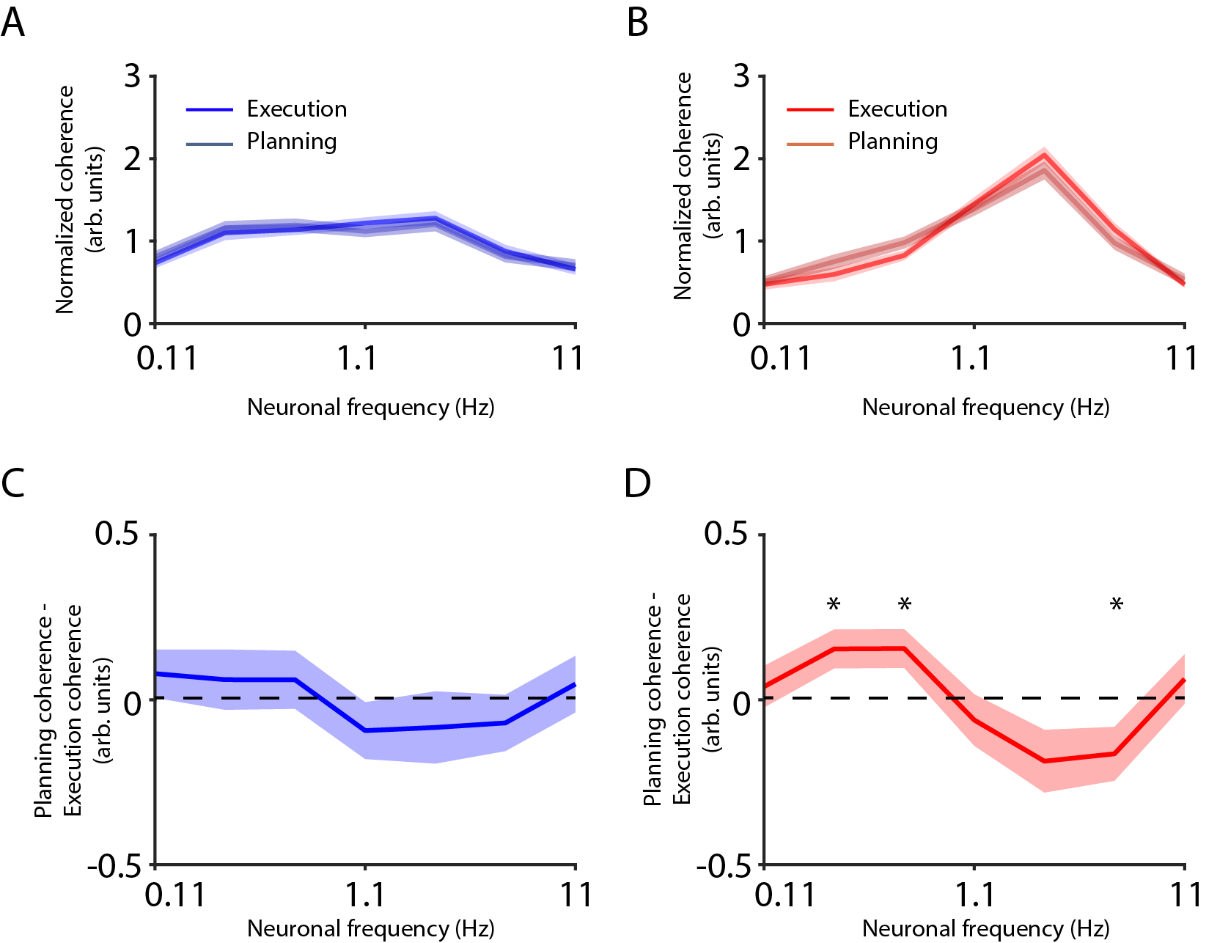


**Supplementary Figure 11.** Frequency specific planning coherence. A: Coherence between the local field potential and the neuronal activity for the locomotor task (dark blue = planning, light blue = execution). The frequency of the neural activity was calculated from the total spiking activity across all electrodes. The shaded areas indicate the standard deviation of the mean. B: Same as in A, but for the joystick task (dark red = planning, light red = execution). C: Difference between the coherence for the planning and for the execution for the locomotor task. The shaded areas indicate the standard deviation of the mean. Two-sided t-test (n=78). No correction for multiple comparisons. D: Same as in C, but for the joystick task. Two-sided t-test (n=78), p=0.0108, 0.0098, and 0.048. No correction for multiple comparisons. Significances are indicated according to * p < 0.05. Source data are provided as a Source Data file.


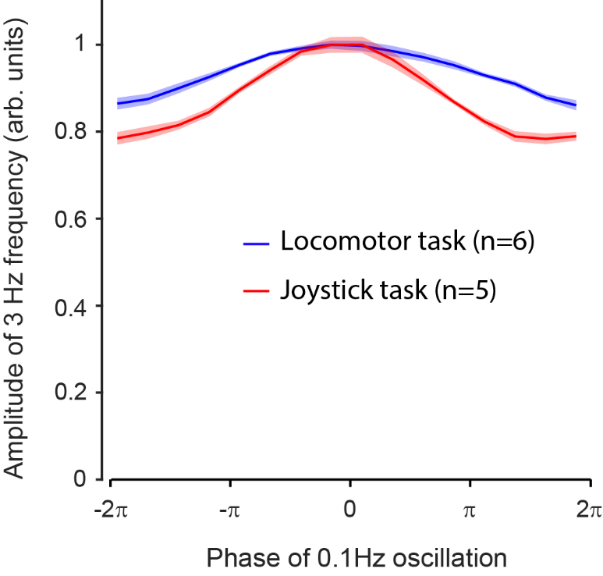


**Supplementary Figure 12**: Phase dependent amplitude of modulation of neuronal activity. The instantaneous amplitude (y-axis) of a 3Hz bandpass filtered neuronal activity was averaged according to the phase (x-axis) of a 0.1 Hz bandpass filtered neuronal activity for the locomotor task (blue) and for the joystick task (red). The solid lines denote the mean across the animals, and the shaded area denotes the standard deviation of the mean across the animals (n=5 for joystick task, 6=for locomotor task). Source data are provided as a Source Data file.

**Supplementary Table 1**

| Animal | SU  mod | SU  all | MU  mod | MU  all | SU+MU  mod | SU+Mu  all | Session count |
| --- | --- | --- | --- | --- | --- | --- | --- |
| 148 | 157 | 530 | 442 | 800 | 599 | 1330 | 14 |
| 220 | 217 | 572 | 477 | 834 | 694 | 1406 | 13 |
| 221 | 105 | 595 | 382 | 972 | 487 | 1567 | 18 |
| 222 | 367 | 1321 | 679 | 1566 | 1046 | 2887 | 21 |
| 223 | 726 | 1899 | 929 | 1787 | 1655 | 3686 | 21 |
| 224 | 253 | 774 | 679 | 1366 | 932 | 2140 | 20 |
| Sum | 1825 | 5691 | 3588 | 7325 | 5413 | 13016 | 107 |

Counts of neuronal units recorded during the locomotor task. mod - modulated units.

**Supplementary Table 2**

| Animal | SU  mod | SU  all | MU  mod | MU  all | SU+MU  mod | SU+MU  all | Session count |
| --- | --- | --- | --- | --- | --- | --- | --- |
| 148 | 76 | 649 | 236 | 863 | 312 | 1512 | 15 |
| 220 | 0 | 0 | 0 | 0 | 0 | 0 | 0 |
| 221 | 56 | 787 | 227 | 1059 | 283 | 1846 | 20 |
| 222 | 185 | 777 | 547 | 1181 | 732 | 1958 | 15 |
| 223 | 270 | 1198 | 609 | 1193 | 879 | 2391 | 15 |
| 224 | 97 | 630 | 308 | 972 | 405 | 1602 | 15 |
| Sum | 684 | 4041 | 1927 | 5268 | 2611 | 9309 | 80 |

Counts of neuronal units recorded during the joystick task. mod - modulated units.

**Supplementary Table 3**

| **Fig.** | **Groups:**  **a** refers to animal count,  **u** refers to sorted unit count,  **u_c_** = u/7 refers to corrected unit count used for significance | **Statistical Analysis** |
| --- | --- | --- |
| Fig. 1l | Locomotor task, Motor-Sensory, (u = 5413, u_c_=773)  Joystick task, Motor-Sensory, (u = 2611, u_c_ = 373) | Two-tailed paired t-test, *P* < 0.0001  Two-tailed paired t-test, *P* < 0.0001 |
| Fig. 2d | Locomotor task, Absolute lag, (u = 5413, u_c_=772)  Locomotor task, Duration (6 bins), (u = 5413, u_c_=772) | RM One-Way ANOVA, F (5, 776) = 474, *P* < 0.0001 |
| Fig. 2d | Joystick task, Absolute lag, (u =2611, u_c_ = 373)  Joystick task, Duration (6 bins), (u =2611, u_c_ = 373) | RM One-Way ANOVA, F (5, 114) = 72, *P* < 0.0001 |
| Fig. 2d (left) | Locomotor task, S1 Duration, (u = 1766, u_c_=252)  Locomotor task, M1 Duration, (u = 1197, u_c_=350)  Locomotor task, M2 Duration, (u=1197, u_c_=171) | RM One-Way ANOVA, F (2, 770) = 171, *P* < 0.0001  Post Hoc: No Bonferroni:  S1-M1: p=0.0021, Mixed effect: p=0.23  M1-M2: p=0.024, Mixed effect: p<0.0001  S1-M2: p<0.0001, Mixed effect: p<0.0001 |
| Fig. 2d (right) | Joystick task, S1 Duration, (u = 837, u_c_=120)  Joystick task, M1 Duration, (u = 1015, u_c_=145)  Joystick task, M2 Duration, (u= 759, u_c_=108) | RM One-Way ANOVA F (2, 370) = 98, *P* < 0.0001  Post Hoc: No Bonferroni:  S1-M1: p<0.0001, Mixed effect: p=0.015  M1-M2: p<0.0001, Mixed effect: p=0.014  S1-M2: p<0.0001, Mixed effect: p<0.0001 |
| Fig. 3f | Locomotor task, Relative time constant, (a = 6)  Joystick task, Relative time constant (a = 5) | Two-tailed t-test, *P* = 0.20  Two-tailed t-test, *P* = 0.043 |
| Fig. 3h | Locomotor task, Absolute time constant, (a = 6)  Joystick task, Absolute time constant (a = 5) | RM One-Way ANOVA, F (3, 18) = 6.6, *P =* 0.0033  Post Hoc: 4-way Bonferroni:  Lowest paw velocity in the Joystick task vs Highest paw velocity in the Locomotor task: p = 0.0348  Lowest paw velocity in the Joystick task vs Highest paw velocity in the Joystick task: p = 0.0108 |
| Fig. 4d | Locomotor task, low-pass versus high-pass, Pearson correlation, (a = 6)  Joystick task, low-pass versus high-pass, Pearson correlation, (a = 5) | Two-tailed t-test, *P* = 0.016  Two-tailed t-test, *P* = 0.0091 |
| Fig. 4d | Locomotor task, high-pass, Lag, (a = 6)  Joystick task, high-pass, Lag, (a = 5) | Two-tailed t-test, *P* = 0.33  Two-tailed t-test, *P* = 0.01 |
| Fig. 4d | Locomotor task, low-pass, Lag, (a = 6)  Joystick task, low-pass, Lag, (a = 5) | Two-tailed t-test, *P* = 0.0013  Two-tailed t-test, *P* = 0.0029 |
| Fig. 4e | Locomotor task, Pearson correlation, (a = 6) | RM One-Way ANOVA F (6, 35) = 3.6, *P =* 0.007  Post Hoc (vs peak at 1,1Hz, no Bonferroni correction):  0.11 Hz: Two tailed t-test, P=0.016  0.23 Hz: Two tailed t-test, p=0.0029  0.45 Hz: Two tailed t-test, p=0.019 |
| Fig. 4e | Joystick task, Pearson correlation, (a = 6) | RM One-Way ANOVA F (6, 28) = 3.5, *P =* 0.0104  Post Hoc (vs peak at 2.3Hz, no Bonferroni correction):  0.11 Hz: Two tailed t-test, p=0.036  0.23 Hz: Two tailed t-test, p=0.012  0.45 Hz: Two tailed t-test, p=0.0085  1.1 Hz: Two tailed t-test, p=0.021 |
| Fig. 4f | Locomotor task, Lag, (a = 6) | RM One-Way ANOVA F (6, 35) = 5.8, *P =* 0.00026  Post Hoc (vs 0 lag, no Bonferroni correction):  0.11 Hz: Two tailed t-test, p=0.0013  0.23Hz: Two tailed t-test, p=0.045 |
| Fig. 4f | Joystick task, Lag, (a = 6) | RM One-Way ANOVA F (6, 28) = 2.0, *P =* 0.1  Post Hoc (vs 0 lag, no Bonferroni correction):  0.11 Hz: Two tailed t-test, p=0.0051  0.23 Hz: Two tailed t-test, p=0.025  1.1 Hz: Two tailed t-test, p=0.0056  2.3 Hz: Two tailed t-test, p=0.005  4.5 Hz: Two tailed t-test, p=0.0072  11Hz: Two tailed t-test, p=0.012 |
| Fig. 5j | Locomotor (n=57 with encoded response larger than 5) versus Joystick task (n=110 with encoded response larger than 5), Latency variability | Boostrap, 1000 rep, P=0.011, corrected unit count u/7. |
| Fig. 6b | High frequency, State coding, Joystick task (n=9439 time points, n_c_=940)  Low frequency, State coding, Joystick task (n=5837 time points, n_c_=580) | Two-tailed t-test, P=0.033, Data sample dependence correction for the temporal smoothing of 10 samples.  Two-tailed t-test, P=0.04, Data sample dependence correction for the temporal smoothing of 10 samples. |

**Supplementary Note 1:** The difference in time constants of the population activity cannot be explained by behavioral differences across the two tasks

Could the differences in time constants of the population activity be explained by differences in behavior across the two tasks? To address this question, we tested whether the autocorrelation of two easily accessible behavioral parameters can explain the observed effects: (1) paw velocity and (2) egocentric paw position. As the population correlation decayed more slowly in the locomotor task, we would expect a temporally broader behavioral autocorrelation for the locomotor task compared to the joystick task. However, the similarly narrow peaks of the autocorrelation of the paw velocity in both tasks (Supplementary Fig. 4A, middle panel row) argue that the difference in the time constant of the population correlation (Supplementary Fig. 4A, upper panel row) cannot be explained by differences in paw velocity. Similarly, for the paw position, we would expect a temporally broader behavioral autocorrelation for the locomotor task compared to the joystick task to explain the differences of the time constants of the population activity. Instead, the autocorrelation of the paw position was narrower during the locomotor task than during the joystick task (Supplementary Fig. 4A, lower panel row). Thus, neither the velocity autocorrelation, nor the position autocorrelation, could explain the differences in the time constants of population activity. Alternatively, neurons preferentially encode the position during the locomotor task, and the velocity during the joystick task. If this were true, the broad population correlation during locomotion could be explained by the broad position autocorrelation and the narrow population correlation during the joystick task could be explained by the narrow velocity autocorrelation. To this end, we tested the encoding preference (position or velocity) of the neurons in the two tasks. The neurons showed an encoding preference for position in the joystick task (Supplementary Fig. 4B), which stands in contrast to the more precise auto-correlation for paw velocities in the joystick task. These opposing results suggest that differences in the time constant of population activity cannot be explained by a differential encoding preference of position and velocity. To summarize, there is a strong decorrelation during paw movements in the joystick task, which cannot be explained by means of differences in behavioral statistics.

**Supplementary Note 2:** Adaptation mechanisms and compatibility with prolonged movements

Adaptation mechanisms at any stage between the cortex and the muscles could serve as the biological equivalent of a high pass filter. The high pass filter should detect fast changes in the activity. On the level of neuronal spiking this can be a change from a high firing rate to a low firing rate, or vice versa. On the level of summed synaptic input this can be the change from a large input current or a low input current, or vice versa. There are numerous neuronal phenomena that describe high-pass filtering on the time scale of hundreds of milliseconds, such as spike rate adaptation^1^, short term synaptic depression^2^, integrating inhibitory neurons^3^, low-pass filtering across gap junction connected interneurons^4^, and depolarization block^5^. The underlying mechanisms of those phenomena can follow the slowly evolving motor planning and sensory integration activity by means of the intracellular calcium concentration, amount of release ready vesicles in the presynaptic terminal, the firing rate of integrating inhibitory neurons, or the number of inactivated sodium channels, respectively.

The generation of prolonged movements under the control of an adaptation related high pass mechanism would require a subcortical process that can be triggered by short lasting inputs. In the lamprey, the reticulospinal cells transform a short duration sensory input into a long-lasting excitatory command^6^. In the zebrafish, high frequency stimulation in the brain stem initiates sustained locomotor behavior^7^. Similarly, in the basal ganglia of rodents, neurons are activated during the initiation and termination of movement sequences^8^. In the mouse, the lower pyramidal tract neurons have been shown to have a preference for motor execution^9^ and are thus good candidates for contributing to sustaining movements. These neuronal processes in combination with our proposed high pass filtering mechanisms would allow for movements of different durations.

**References**

1. Baldissera, F., Gustafsson, B. & Parmiggiani, F. Saturating summation of the afterhyperpolarization conductance in spinal motoneurones: a mechanism for ‘secondary range’ repetitive firing. *Brain Res.* **146**, 69–82 (1978).

2. Zucker, R. S. & Regehr, W. G. Short-term synaptic plasticity. *Annu. Rev. Physiol.* **64**, 355–405 (2002).

3. Silberberg, G. & Markram, H. Disynaptic inhibition between neocortical pyramidal cells mediated by Martinotti cells. *Neuron* **53**, 735–746 (2007).

4. Coulon, P. & Landisman, C. E. The Potential Role of Gap Junctional Plasticity in the Regulation of State. *Neuron* **93**, 1275–1295 (2017).

5. Bianchi, D. *et al.* On the mechanisms underlying the depolarization block in the spiking dynamics of CA1 pyramidal neurons. *J. Comput. Neurosci.* **33**, 207–225 (2012).

6. Dubuc, R. *et al.* Initiation of locomotion in lampreys. *Brain Res. Rev.* **57**, 172–182 (2008).

7. Kyriakatos, A. *et al.* Initiation of locomotion in adult zebrafish. *J. Neurosci. Off. J. Soc. Neurosci.* **31**, 8422–8431 (2011).

8. Jin, X. & Costa, R. M. Shaping Action Sequences in Basal Ganglia Circuits. *Curr. Opin. Neurobiol.* **33**, 188–196 (2015).

9. Economo, M. N. *et al.* Distinct descending motor cortex pathways and their roles in movement. *Nature* **563**, 79–84 (2018).
