# Supplementary material for: Distinct dynamics of neuronal activity during concurrent motor planning and execution
Source: Nat Commun. 2021 Sep 10;12:5390. doi: 10.1038/s41467-021-25558-8 (PMC8433382; doi:10.1038/s41467-021-25558-8)
Supplement: Supplementary file 2 — Description of Additional Supplementary Files [file 41467_2021_25558_MOESM2_ESM.pdf]

Description of Additional Supplementary Files

File Name: Supplementary Movie 1
Description: Effect of Optogenetic Stimulation in CFA on Front Paw movements
